# Oscillatory DeltaC Expression in Neural Progenitors Primes the Prototype of Forebrain Development

**DOI:** 10.1007/s12035-024-04530-9

**Published:** 2024-10-11

**Authors:** Fang-Shin Nian, Bo-Kai Liao, Yen-Lin Su, Pei-Rong Wu, Jin-Wu Tsai, Pei-Shan Hou

**Affiliations:** 1 Institute of Anatomy and Cell Biology, National Yang Ming Chiao Tung University, Taipei, Taiwan; 2 Institute of Clinical Medicine, College of Medicine, National Yang Ming Chiao Tung University, Taipei, 112 Taiwan; 3 Department of Aquaculture, National Taiwan Ocean University, Keelung, Taiwan; 4 Institute of Brain Science, College of Medicine, National Yang Ming Chiao Tung University, Taipei, 112 Taiwan; 5 Brain Research Center, National Yang Ming Chiao Tung University, Taipei, Taiwan; 6 Department of Biological Science and Technology, College of Biological Science and Technology, National Yang Ming Chiao Tung University, Hsinchu, 300 Taiwan

**Keywords:** Notch signaling, Delta gene, Forebrain development, Oscillation pattern

## Abstract

Notch signaling plays a pivotal role in regulating various developmental processes, particularly in controlling the timing of neuronal production within the developing neocortex. Central to this regulatory mechanism is the oscillatory pattern of Delta, which functions as a developmental clock modulator. Its deficiency profoundly impairs mammalian brain formation, highlighting its fundamental role in brain development. However, zebrafish carrying a mutation in the functional ortholog DeltaC (dlc) within their functional ortholog exhibit an intact forebrain structure, implying evolutionary variations in Notch signaling within the forebrain. In this study, we unveil the distinct yet analogous expression profiles of *Delta* and *Her* genes in the developing vertebrate forebrain. Specifically, for the first time, we detected the oscillatory expression of the *Delta* gene *dlc* in the developing zebrafish forebrain. Although this oscillatory pattern appeared irregular and was not pervasive among the progenitor population, attenuation of the dlc-involved Notch pathway using a γ-secretase inhibitor impaired neuronal differentiation in the developing zebrafish forebrain, revealing the indispensable role of the dlc-involved Notch pathway in regulating early zebrafish neurogenesis. Taken together, our results demonstrate the foundational prototype of dlc-involved Notch signaling in the developing zebrafish forebrains, upon which the intricate patterns of the mammalian neocortex may have been sculpted.

**Supplementary Information:**

The online version contains supplementary material available at 10.1007/s12035-024-04530-9.

## Introduction

Sensory processing in the vertebrate forebrain allows us to react to environmental stimuli. Although the neural induction process, such as neural tube formation, is generally conserved, the cell alignment in tetrapod forebrain differs from that in zebrafish forebrain [1]. In the developing tetrapod forebrain, the neural progenitors reside on the shore of ventricles within a pipe-like structure named the ventricular zone, while neural progenitors sit on the coast of the dorsal sector with a stream spreading to the medial region, forming a T-shaped germinal zone in the developing forebrain of zebrafish. Despite their place of residence, these progenitors serve as a source for producing neurons. The generated neurons migrate in a species-specific manner toward the final destination and form connections to establish sensory circuits. Although the cytoarchitecture may vary among vertebrates, a conserved molecular expression profile has been identified in multiple species, such as progenitor genes *Pax6*, *Hes1*, and *Sox2*; intermediate progenitor gene *Eomesa/Tbr2*; and neuronal gene *HuC* [1–4].

The conserved molecular expression profile suggests a preserved molecular mechanism for regulating neurogenesis. Notch signaling is critical in regulating the transition of neuroepithelial cells to radial glial cells and the differentiation of radial glial cells to intermediate progenitors or neurons [5–7]. Dll1, the primary Notch ligand in mammals, interacts with Notch receptors on the signal-receiving cells to deliver Notch signaling. Dll1-Notch interaction triggers the release of the Notch intracellular domain (NICD), which activates the transcription of downstream genes, such as the progenitor gene *Hes1*. In the signal-receiving cells, Hes1 suppresses the expression of the proneuronal gene *Ngn2* to maintain progenitor identity and prevent neuronal differentiation. The downregulation of Ngn2, in turn, leads to a decrease in Dll1 expression, which is opposed to the signal-sending cells. The reciprocal expression of Dll1 and Hes1 in the neighboring cells is known as lateral inhibition [8]. While this simple and one-directional regulation is known to be the primary Notch signaling pathway in invertebrates, such as drosophila [9], additional crosstalk among cells and complicated regulation of effector protein metabolism have been found to modulate the Notch signaling within a population in the developing complex tissues of vertebrates, such as the somite, and in mammals, such as the forebrains [10, 11]. In mammalian cortical progenitors, the downregulation of Dll1 effector gene Hes1, due to its self-transcriptional suppression and a short half-life, induces the upregulation of the proneuronal gene *Ngn2*, which in turn activates the downstream gene *Dll1*, leading to the induction of Hes1 expression in the neighboring cells. As a consequence, the expression of Dll1, Hes1, and Ngn2 oscillate in undifferentiated cortical progenitors before sustained high Ngn2 expression drives neural differentiation [12]. Nevertheless, it remains unclear why the dynamics of Notch signals change during evolution and what the relationship is between the complicated oscillated Notch signal gene expression pattern and neurogenesis.

Even though the molecular mechanism of neurogenesis during the development of pallium in larval zebrafish remains unclear, in situ hybridization and histochemistry have shown that neural progenitor cells in larval zebrafish pallium share the same molecular markers as those in other species and are generated primarily in the T-shaped germinal region in the forebrain [13]. Moreover, neural progenitors in adult neurogenesis express similar molecular markers in a conserved location in the forebrain of adult zebrafish, and the molecular mechanisms of adult neurogenesis have been well-studied in both zebrafish and rodents. These studies have yielded insights into the development of the pallium in zebrafish, providing knowledge and potential avenues of investigation. While embryonic neural progenitors in mammals reside along a wide range of germinal zones, the adult neurogenesis region is restricted to a few places, such as the hippocampus and subventricular zone. In contrast, neural progenitors in teleost brains can be found in many areas during both larval and adult stages [14, 15]. Despite differences in the number and location of neural progenitors in zebrafish and mammalian brains, both use the Notch signaling pathway to maintain the progenitor pool and select the quiescent neural stem cells for adult neurogenesis [12, 16–18]. In mammals, the dynamics of Notch1 signaling components, such as Hes1 and Dll1, are critical to determining the status of neural stem cells. For example, embryonic forebrain progenitors and active adult neural stem cells exhibit an oscillatory expression pattern, while quiescent adult neural stem cells have a sustained expression pattern [11, 18]. However, it is currently unknown whether the expression profile of the Notch signal in the developing zebrafish forebrain and its roles in regulating neurogenesis is conserved in mammals.

**Fig. 1:**
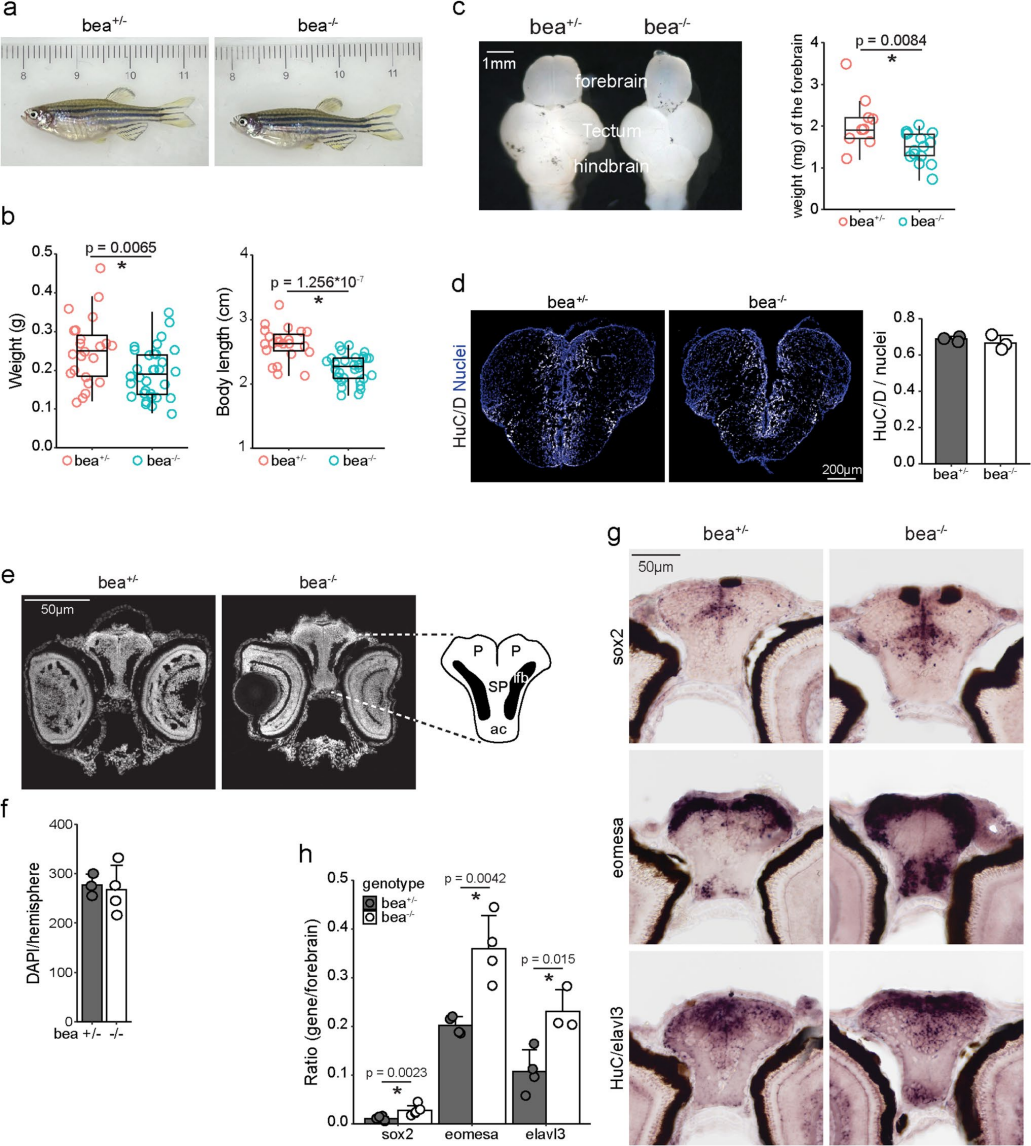
Increased neurons and progenitors in the forebrain of *dlc* mutant larval zebrafish but no gross abnormality in adults. **a** Image of *dlc* mutant and control adult zebrafish. **b** Quantitative analysis of body index of adult zebrafish. **c** Image and quantitative analysis of forebrain in *dlc* mutant and control adult zebrafish. **d** Image and quantitative analysis of the ratio of HuC/D-positive cells to total cells (nuclei) in adult zebrafish forebrains. Nuclei: DAPI staining. **e, f** Image and quantitative analysis of the number of nuclei in the forebrains of 5 dpf larval zebrafish. Nuclei: DAPI staining. P, pallium; SP, subpallium; lfb, lateral forebrain bundle; ac, anterior commissure [13]. **g, h** In situ hybridization and quantitative analysis of larval zebrafish at 5 dpf using probes specifically targeting progenitor gene *sox2*, neuronal precursor gene *eomesa*, and neuronal gene *HuC/elavl3*. Scale bars are indicated. Error bars represent standard deviation, and data points are shown in dots. * Significance with *p* value < 0.05 by Student’s *t*-test

Supporting the critical and sophisticated regulated Notch signaling, aberrant expression of Hes1 can induce severe brain malformation in mammals [19, 20], and *DLL1* point mutation in humans induces nonspecific brain abnormalities with or without seizures (NEDBAS) [21]. Surprisingly, zebrafish carrying the *Delta* mutation survived with intact forebrain morphology compared to the controls, suggesting the evolving characteristics of Notch signaling in forebrain development (Fig. 1a, b). In this study, we examined the spatial expression pattern of *Delta* and *Her* genes in the developing forebrain of zebrafish, turtles, chickens, and mice. We found similar but variable distribution patterns of *Delta*-positive cells in the developing forebrains of zebrafish compared to chickens and mice. Additionally, the oscillatory expression pattern of the *Delta* gene, *DeltaC/dlc*, was detected and monitored for the first time in the developing zebrafish forebrain. Although the effects of attenuating dlc-involved Notch signaling on neuronal differentiation in the developing zebrafish forebrain were inconsistent with those in chickens and mice, the irregular dlc expression profile in zebrafish suggested a foundational prototype of dlc-involved Notch signaling in regulating forebrain development. These results demonstrate the convergent evolution of Notch signaling in the telencephalon, which would ultimately provide the fundamental basis for establishing the cortex/neocortex for complex sensory processing.

## Materials and Methods

### Animals

ICR wild-type mice, *Mus musculus*, were used in this study. The day of vaginal plug detection was defined as embryonic day (E) 0.5 and the day of birth as postnatal day (P) 0. Mice were hosted at the National Yang Ming Chiao Tung University Laboratory Animal Center following the instructional guidelines. Stocks of adult zebrafish, *Danio rerio*, were kept at the fish facilities of National Ocean University in 28 °C fresh water under a 14:10 h light:dark photoperiod, following the instructional guidelines. Embryos were bathed in Petri dishes with E3 media within 30 min after fertilization and maintained at 28.5 °C. Eggs of fertilized Chinese soft-shelled turtle, *Pelodiscus sinensis*, were provided by a turtle farm in Taoyuan City, Taiwan. The eggs were incubated in wood scraps in styrene foam boxes at 30 ± 2 °C, with moisture provided occasionally. The developmental stage of *P. sinensis* was determined as described previously [22]. Fertilized chicken eggs were provided by the Animal Health Research Institute, Council of Agriculture, Taiwan. The chicken eggs were incubated in an incubator at 37 °C with humidity maintained at 60–80%.

### Sample Collection of *bea*^*tm98*^Mutant Zebrafish

The zebrafish strain with *dlc/bea*^*tm98*^ mutation was used in this study [23]. Homozygous mutant *dlc/bea*^*tm98/tm98*^ fish are denoted as *bea*^*−/−*^, and heterozygous *dlc/bea*^*tm98/*+^ fish are denoted as *bea*^*+/-*^. To obtain both *bea*^*−/−*^ and *bea*^*+/-*^ larval zebrafish, we set up mating using one *bea*^*−/−*^ and one *bea*^*+/-*^ fish and collected the fertilized eggs. At 12–20 h post-fertilization, *bea*^±^ fishes were distinguished based on the completeness of somite, which is disrupted in *bea*^*−/−*^ fishes.

To collect samples for ISH analysis, bea^−/−^ and bea^+/-^ zebrafish were fixed in 4% paraformaldehyde at 4 °C overnight and then transferred to ice-cold methanol at − 20 °C for more than 2 h for permeabilization. Before cryosectioning, samples were immersed in 30% sucrose–PBS at 4 °C overnight and then embedded in Shandon Cryomatrix frozen embedding medium (Thermo Fisher). Then, 12 µm coronal sections were prepared using a cryostat (NX70; Thermo Fisher) and stored at − 80 °C before ISH or IHC analyses.

### In Situ Hybridization (ISH) Analysis

Digoxigenin (DIG)-labeled antisense probes were prepared, and in situ hybridization was performed as previously described [24]. *dlc* and *dld* ISH probes were obtained from Professor Liao at NTOU. Primers listed in Supplementary Table I were used to amplify the mRNA sequence of indicated genes. After confirmation of PCR product size and sequence, PCR products were linked with the T7 promoter at 3′ end and Sp6 promoter at 5′ end using T7 promoter linker primer (TAATACGACTCACTATAGGGCCCGGGGC) and Sp6 promoter linker adapter primer (ATTTAGGTGACACTATAGAAGGCCGCGG). Digoxigenin (DIG)-labeled probes were transcribed in vitro using T7 and Sp6 promoter-tagged PCR products following the procedure with T7 or Sp6 polymerase. Briefly, samples were pretreated and hybridized with 200–500 ng/mL of probes at 55 °C overnight. Peroxidase-tagged anti-DIG antibody (1:500, Roche) followed by tyramide signal amplification was applied. Signals were developed with Texas Red streptavidin (1:500 vector) at room temperature, followed by DAPI nuclear staining or nitro blue tetrazolium (NBT) and 5-bromo-4-chloro-3-indolyl-phosphate (BCIP) substrates (Roche) at room temperature. In two-color in situ hybridization, the signals were developed by streptavidin conjugated with Alexa Fluor 488/594 (Thermo Fisher). Images were acquired by an IX83 inverted microscope (Olympus). The ISH signals were analyzed using the bulti-in functions in Fiji/ImageJ software (https://imagej.nih.gov/ij/docs/guide/user-guide.pdf). A procedural example is shown in Fig. S11 using a section of a 5 dpf larval zebrafish with Sox2 staining (from Fig. 1g). Begin the image analysis process by opening your original ISH image (Fig. S11a) and defining the region of interest (ROI) intended to analyze, ensuring that this ROI is recorded in the “ROI manager” (Fig. S11a’). Next, convert the original image into an 8-bit format and invert it, which transforms the positive ISH signals into white particles set against a black background (Fig. S11b). To effectively differentiate actual signals from background noise, apply a Gaussian blur to the inverted image, choosing a blur radius between 3 and 5 µm, optimized for the specific RNA probe to ensure that genuine positive signals are rendered invisible under this condition (Fig. S11b’). Now, use the “Image calculator” to subtract the blurred image (Fig. S11b') from the inverted image (Fig. S11b), resulting in a processed image (Fig. S11b”). Afterward, convert the processed image (Fig. S11b”) into a binary format using the “Threshold” function, adjusting the threshold appropriately for the RNA probe to ensure that true positive signals appear distinctly white against a black background (Fig. S11c-c’). Finally, reapply the initially recorded ROI (Fig. S11a’) onto the thresholded image (Fig. S11c’) and employ the “Analyze Particles” feature to determine the ROI of all detected particles. For verification purposes, overlay this ROI on the original image, ensuring alignment with observed positive signals (Fig. S11d-d’). This stepwise process facilitates the accurate analysis of ISH images. The ratio of positive signal area to forebrain area was calculated and is shown as “Ratio (gene/forebrain)” in the bar graphs (Figs. 1h, 5c, and S4b).

### Immunohistochemistry (IHC) Analysis

Immunohistochemistry was performed as previously described [25]. Briefly, 12 μm cryosections were washed with 0.05% Tween-20 in PBS and then blocked with 10% normal donkey serum, 0.2% Tween-20, and 0.1% Triton X-100 in PBS at room temperature for 1 h. The primary antibodies were applied in 1% normal donkey serum in PBS at 4 °C overnight. After washing with 0.05% Tween-20 in PBS, secondary antibodies were applied in 1% normal donkey serum in PBS at room temperature for 1 h. Nuclei were counterstained with DAPI solution (Thermo Fisher). Images were acquired using an IX83 fluorescence microscope (Olympus) and an LSM700 confocal microscope (Carl Zeiss). The following primary antibodies were used: mouse anti-HuC/D (1:500, Millipore), rabbit anti-Tbr2 (1:500, Abcam), and mouse anti-Tuj1 (1:1000, Abcam). Secondary antibodies conjugated with Alexa Fluor dye were used at 1:500 (Thermo Fisher).

### Analysis of Dll1 and Dlc Signals in Developing Brains of Zebrafish, Chickens, and Mice

Fluorescence in situ hybridization images developed with Texas Red streptavidin were acquired using a Zeiss LSM700 confocal microscope. Cells and signals within cells were determined using the Mastodon package by J.V. Tinevez (https://github.com/mastodon-sc/mastodon) in Fiji/ImageJ software [26, 27]. Differences in the Gaussian detector were used for edge detection. Cell radii were determined to include the majority of cells with intact nuclei, and the threshold of signal intensity was determined in order not to include background signals. DAPI signal was used as an internal control to rectify the *Delta* signal. Based on the signal distribution of Delta/DAPI using a histogram diagram, a threshold was set to define *Delta*-positive cells. The minimal distance between a cell and the nearest cell and between a signal-positive cell and the nearest signal-positive cell was calculated using Excel software. Then, the relative minimal distance between signal-positive cells and the nearest signal-positive cells was calculated by dividing the minimal distance between a signal-positive cell and the nearest signal-positive cell by the minimal distance between a cell and the nearest cell. Ripley’s K function was adapted to perform spatial point analysis following previously published methods [28, 29]. In brief, the L function, L(), is the linear solution of the K function to reveal clustered, random, or regular distributions of points by sampling point counts with gradually enlarged circles. Since the brain slices were relatively thin in the Z-axis, XY projections in 2D were used for calculating L() in order to prevent overly clustered outputs. The areas of L() were defined by the minimal ranges of X- or Y-coordinates, and 100-gradated radii of the areas were used for each slice. The artificial *Delta* patterns of most clusters or regular distributions were generated with the corresponding numbers of *Delta*-positive cells in each slide as the controls to show the extreme distributions and 80 random Delta distributions were generated to calculate the 99% confidence intervals.

### Inhibition of Notch Signaling

For mice, brain slices were prepared and cultivated as previously described [30]. Then, 500 µm coronal E13.5 mouse brain slices were prepared using a vibratome (Leica) and then were placed on Millicell-CM inserts (Millipore) in culture medium containing 25% Hanks’ balanced salt solution (Sigma Aldrich) and 60% basal MEM supplemented with 5% FBS, 1% N-2 Supplement, 1% penicillin/streptomycin, 1% L-glutamine (Gibco), and 0.66% glucose. For chicken, 300 µm coronal brain slices were prepared using a vibratome (Leica) and then were placed on Millicell-CM inserts (Millipore) in culture medium containing Neurobasal Medium (Gibco) supplemented with 5% horse serum, 1% N-2 Supplement, 2% B-27 Supplement, 0.5% penicillin/streptomycin, 1% L-glutamine (Gibco), and 1% 1 M HEPES–NaOH (pH 7.3) (1 mL) [31, 32]. For zebrafish, 20–30 larval zebrafishes in the chorions were bathed in one well of 6-well plate with E3 medium [33]. To inhibit Notch signaling, γ-secretase inhibitor MK-0752 (Cayman Chemical) was applied in the culture medium. Freshly prepared MK-0752 at a concentration of 25 μM in culture medium was applied to mouse and chicken brain slices, and 10 μM MK-0752 in E3 medium was applied to larval zebrafish. DMSO, the solvent of the MK-0752 working solution, was used as the control. The culture condition for mouse cortices was 37 °C for 20 h in a humid incubator with 5% CO_2_; for chicken, cortices were 30 °C for 24 h in a humid incubator containing 5% CO_2_ and 40% O_2_; and for zebrafish was 28.5 °C for 24 h. After treatment, samples were fixed with 1% paraformaldehyde at 4 °C overnight. After immersion in 30% sucrose–PBS at 4 °C overnight, samples were embedded in Shandon Cryomatrix frozen embedding medium (Thermo Fisher). Then, 12 µm sections were prepared using a cryostat (NX70; Thermo Fisher). Sections before ISH or IHC analyses were stored at − 80 °C before ISH or IHC analyses.

### Live Imaging and Analysis of Zebrafish Brains and Somite

For brains, to trace dlc expression within a cell, we injected GFP plasmid into 4-cell-stage zebrafish embryos carrying *Tg(dlc11k:mCherry)*, named dlc-mCherry [34]. 1-Phenyl 2-thiourea (PTU) at a final concentration of 0.003% was applied before 12 h post-fertilization (hpf) to prevent melanogenesis. Before imaging, 2 dpf zebrafish were anesthetized with 50 mg/L tricaine in E3 medium. For somite, the larval zebrafish at the 15–20 somite stage were dechorionated and anesthetized with 50 mg/L tricaine in an E3 medium. To acquire time-series images, embryos were embedded in 1% low-melting agarose and then immersed in an E3 medium with tricaine to maintain the moisture. Images were acquired every 5 min for 5 h at a temperature of 28.5 °C using an LSM880 confocal microscope (Carl Zeiss). To monitor mCherry signals, we first identified and tracked cells using GFP signals and then applied the built-in timelapse/circle interpolator function in the Fiji/ImageJ software. The average intensity of GFP cells was subjected to further analysis to evaluate the mCherry dynamics using MATLAB software with built-in MATLAB functions. As systematic bias, such as the accumulating impacts from laser exposure, may occur during the live imaging process, we first normalized the mean intensity of dlc-mCherry to GFP intensity and then processed the detrending procedure in order to reveal the real dynamics [35]. A trend line signal was calculated by computing the moving mean of the signal with a window size of 100 min using a MATLAB-built-in movmean function and then subtracted from the normalized signal. The signal was smoothed using a moving mean with a 20-min window size. To detect the peaks, a MATLAB-built-in findpeaks function was applied with a threshold set to 0.1 times the dynamic range. To evaluate if the dynamics resemble the oscillated pattern found in mouse neural progenitors [12], we analyzed the autocorrelation structure of the waveform using a MATLAB-built-in autocorr function. The results of 59 lags were calculated from 60 time points. The results were shown in pseudo-color, 16_colors, in a look-up table (LUT).

### Cell Clustering

To cluster the oscillating behaviors, the *k-means* machine learning method was utilized on the *DATAtab* statistics software (https://datatab.net/statistics-calculator/cluster). The input parameters of each cell were the number of completed oscillation cycles, the mean square of the autocorrelation coefficient of total lags, the range of the autocorrelation coefficient, the mean peak-to-peak interval, and the mean amplitude. To calculate phases, the autocorrelation coefficient below 0.3 (no correlation) was regarded as 0 to avoid overestimation from small fluctuations. For all five parameters, the larger the value was, the more robust the oscillator was. The *k* = 3 clusters and epoch = 50 were used for the *k-means* algorithm to cluster the tracked cell into 3 clusters. *k* = 3 was selected by the elbow method, where variance was most decreased in a series of *k* tests. The epoch was set above the iterations when the clustering results were no longer changed, which was approximately epoch ≧ 30 for our data sets.

### Statistical Analysis

The data are shown as dot plots overlaid with either a bar chart representing the mean ± SD or a box-whisker plot representing quartiles. Student’s *t*-test was performed, and significance was recognized at *p* < 0.05.

## Results

### Early Neurogenesis Was Affected in the Forebrain of Dll1 Functional Ortholog *dlc* Mutant Zebrafish

To explore whether loss of Dll1 expression affects brain development in other vertebrates, zebrafish were utilized, as the genome is well studied and genetically manipulated zebrafish are available. In zebrafish, the delta family, containing dla, dlb, dlc, and dld, has been identified as an ortholog of the Dll family in mammals (Fig. S1). While the sequence of Dll1 is highly similar to sequences of dla and dld (Fig. S1), the dynamic expression pattern of Dll1 is different from either of them during embryogenesis. For example, Dll1 is expressed in both somite and brain development in an oscillatory expression pattern [12], while dla is not expressed in the presomitic mesoderm (PSM), and dld is constantly expressed in PSM [36]. Nonetheless, dlc was found to be the only gene of Delta/Serrate/lag-2 (DSL) ligands with oscillating expression in developing somite and serves as the segmentation oscillation modulator during somitogenesis of somite formation in zebrafish [36, 37]. As an oscillatory expression pattern is a critical characteristic of Dll1 in progenitors during cortical development [12], the allied periodic expression patterns of Dll1 and dlc suggest that dlc might be a functional homolog of mammalian Dll1 in zebrafish. We, therefore, examined the effects of the loss of dlc in the brains of zebrafish using *bea*^*tm98*^ zebrafish, named *bea*^*−/−*^, compared to the *bea*^*+/-*^ controls [23].

We found that the zebrafish carrying the homozygous *dlc* mutation survived until the adult stage with normal reproductive ability (Fig. 1a). Nonetheless, the adult *dlc* mutant fish showed smaller body size and lighter body weight (Fig. 1b) as well as lighter forebrain weight compared to the *bea*^*+/-*^ controls (Fig. 1c). To inspect whether the forebrain composition was affected in *dlc* mutants, we prepared 12 µm cryosections for immunostaining and in situ hybridization analysis. The results showed no significant difference in the proportion of HuC/D-positive neurons between adult *dlc* mutants and controls (Fig. 1d), although somite formation was found to be affected at early embryogenesis, as previously reported (Fig. S2a) [38]. To account for the robust regenerative capacity of zebrafish [39], which may obscure the effects of dlc expression loss on early pallium neurogenesis, we analyzed the *dlc* mutant zebrafish at 2 days post-fertilization (dpf) (Fig. S2b) and 5 dpf (Fig. 1e). We found that the number of nuclei in the hemisphere was unaffected based on DAPI staining (Fig. 1f). Nonetheless, the results of in situ hybridization analysis showed that the area where cells expressed the neural progenitor gene *sox2*, intermediate neural progenitor gene *eomesa*, or neuronal gene *HuC/elavl3* was significantly increased (Fig. 1g, h and S3).

Next, we asked whether the minor phenotype was due to compensation for the loss of *dlc* expression by other *delta* genes by examining the expression of all *delta* genes, including *dla, dlb*, and *dld*, as well as *dlc* (Fig. S4). As reported previously [23], at 5 dpf, while *dlc* expression was mildly downregulated in the forebrain of *dlc* mutant (Fig. S4), a severe somite defect was easily observed compared to the control (Fig. S2a), indicating the functional loss of dlc in *bea*^*−/−*^ zebrafish. The expression of *dla* (*p* = 0.086) and *dlb* (p < 0.001) in the forebrain of *dlc* mutant fish was increased, and the expression of *dld* was not changed compared to *bea*^*+/-*^ controls. While the upregulated *dla* and *dlb* expression implies a possible compensation for losing functional dlc expression to activate Notch signaling, the expression of the Notch effector *her6* was significantly decreased (Fig. S4). The downregulated *her6* expression supports the phenotype of prematuration (Fig. 1g, h, and S3) and indicates that the upregulated *dla* and *dlb* expression may not fully compensate for the dlc functions in *dlc* mutant fish. This finding underscores the significant role of dlc in regulating zebrafish forebrain development. Notably, these results are partially in accord with the *Dll1* conditional knocking out phenotype, showing that neuronal gene expression was increased and progenitor gene expression was downregulated in *Nestin-cre*; *Dll1*^*fl/fl*^ cortices, suggesting a phenotype of massive prematuration in the expanse of the progenitor pool [40]. Taken together, our results indicate that the loss of *dlc* would affect neurogenesis in the developing forebrain in a manner similar to the *Dll1*-deficient mammalian neocortex despite no gross abnormality being observed in the adult forebrain.

### Notch Signaling in Forebrain Development Is Not Solely Controlled by Lateral Inhibition Among Vertebrates

**Fig. 2:**
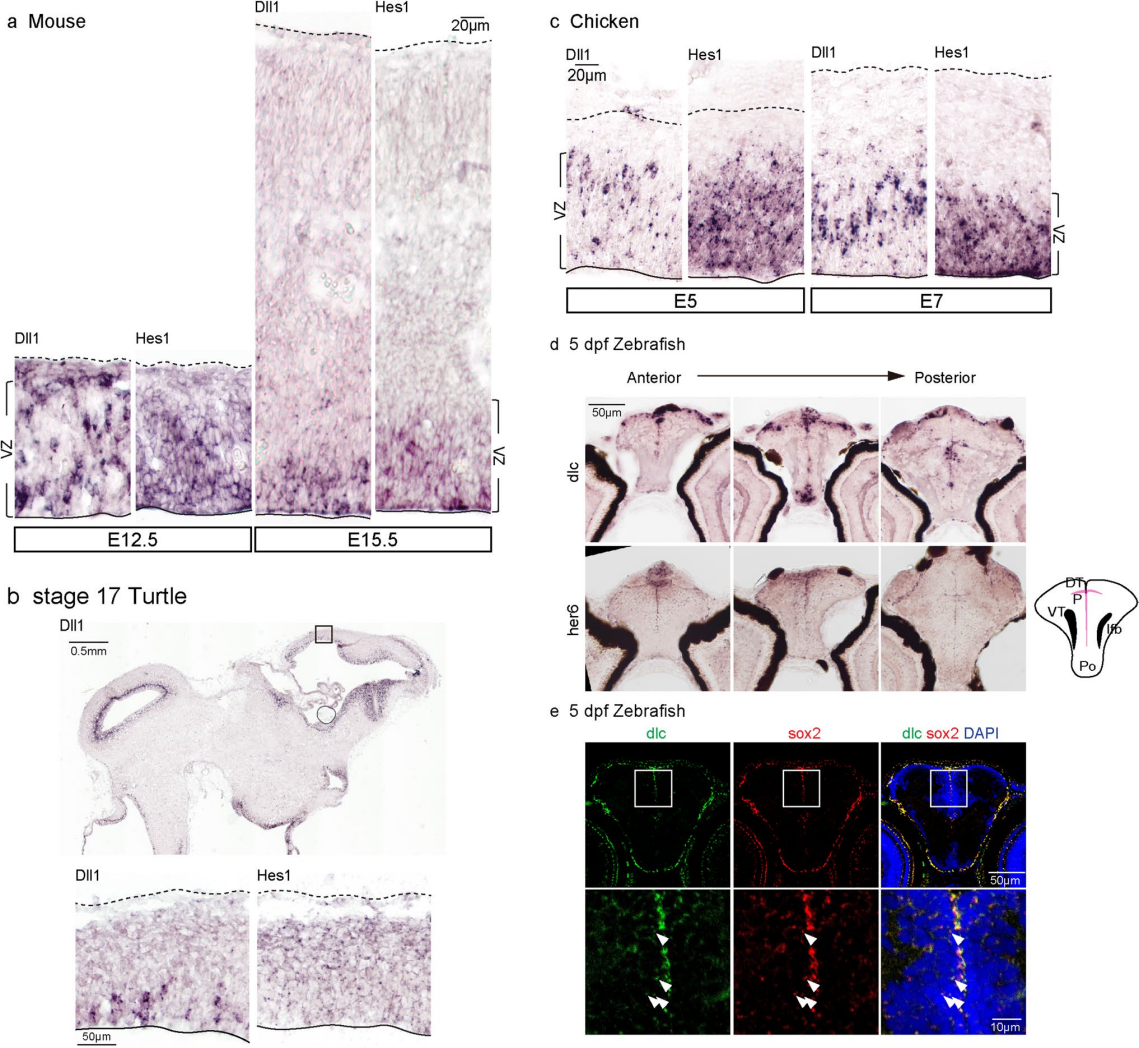
Expression pattern of *Dll1*, *Hes1*, and orthologs in developing vertebral forebrain. **a** In situ hybridization of *Dll1* and *Hes1* in developing mouse dorsal telencephalon at E12.5 and E15.5 using probes specifically targeting mouse *Dll1* and *Hes1* mRNA. **b** In situ hybridization of *Dll1* and *Hes1* in developing dorsal telencephalon of stage 17 turtles using probes specifically targeting turtle *Dll1* and *Hes1* mRNA. **c** In situ hybridization of *Dll1* and *Hes1* in developing chicken dorsal telencephalon at E5 and E7 using probes specifically targeting chicken *Dll1* and *Hes1* mRNA. **d** In situ hybridization of *dlc* and *her6* in developing forebrain of 5 dpf larval zebrafish using probes specifically targeting zebrafish *dlc* and *her6* mRNA. **e** Two-color in situ hybridization of *dlc* and *sox2* in developing forebrain of 5 dpf larval zebrafish. Colocalized signals are indicated by arrowheads. P, pallium; DT, dorsal thalamus (thalamus); VT, ventral thalamus (prethalamus); Po, preoptic region; lfb, lateral forebrain bundle [13]. Scale bars are indicated

Phenotypic differences in Notch signaling deficiency between mice and zebrafish may be due to inconsistent expression patterns of *delta* gene *Dll1* and its orthologs among species, as well as delta transcriptional effector *her gene*, during forebrain development across species. To this, we collected forebrain samples from developing zebrafish and mammalian mice as well as turtles (*Pelodiscus sinensis*) and avians (*Gallus gallus*). In the developing forebrain of larval zebrafish, progenitors were aligned in a T-shaped region [41], contrasting with the ventricular zone observed in tetrapods, including amphibians, reptiles, and mammals (Fig. 2) [42]. In the developing mouse neocortex, *Dll1* was stochastically enriched in the ventricular zone and decreased during later stages, along with *Dll1* transcriptional effector *Hes1* (Fig. 2a) [12]. A similar stochastic expression pattern of *Dll1* could be observed in the developing forebrains of stage 17 turtles and chickens at E5 and 7 (Fig. 2b, c). Furthermore, unlike in mice, *Dll1* and *Hes1* expressions were not downregulated at later stages in chicken brains (Fig. 2c). In larval zebrafish forebrains, *dlc* signals were enriched in the T-shaped germinal region of the pallium and co-expressed with the known neural progenitor gene *sox2*, similar to mouse cortical progenitors [43] (Fig. 2d, e and S4), while *dld*, the sequence-wise ortholog of *Dll1*, was generally expressed in the forebrain, including subdivisions such as pallium and thalamus (Fig. S4 and S5). This conserved specific enriched expression pattern of *dlc* in the germinal zone of zebrafish forebrain, and *Dll1* in the germinal zone of mouse neocortex supports our hypothesis that *dlc* is the functional ortholog of mammalian *Dll1* in zebrafish. Taken together, these results from multiple species show that Dll1 and its orthologs, dlc, are expressed in a similar stochastic pattern in the developing forebrain of vertebrates.

**Fig. 3:**
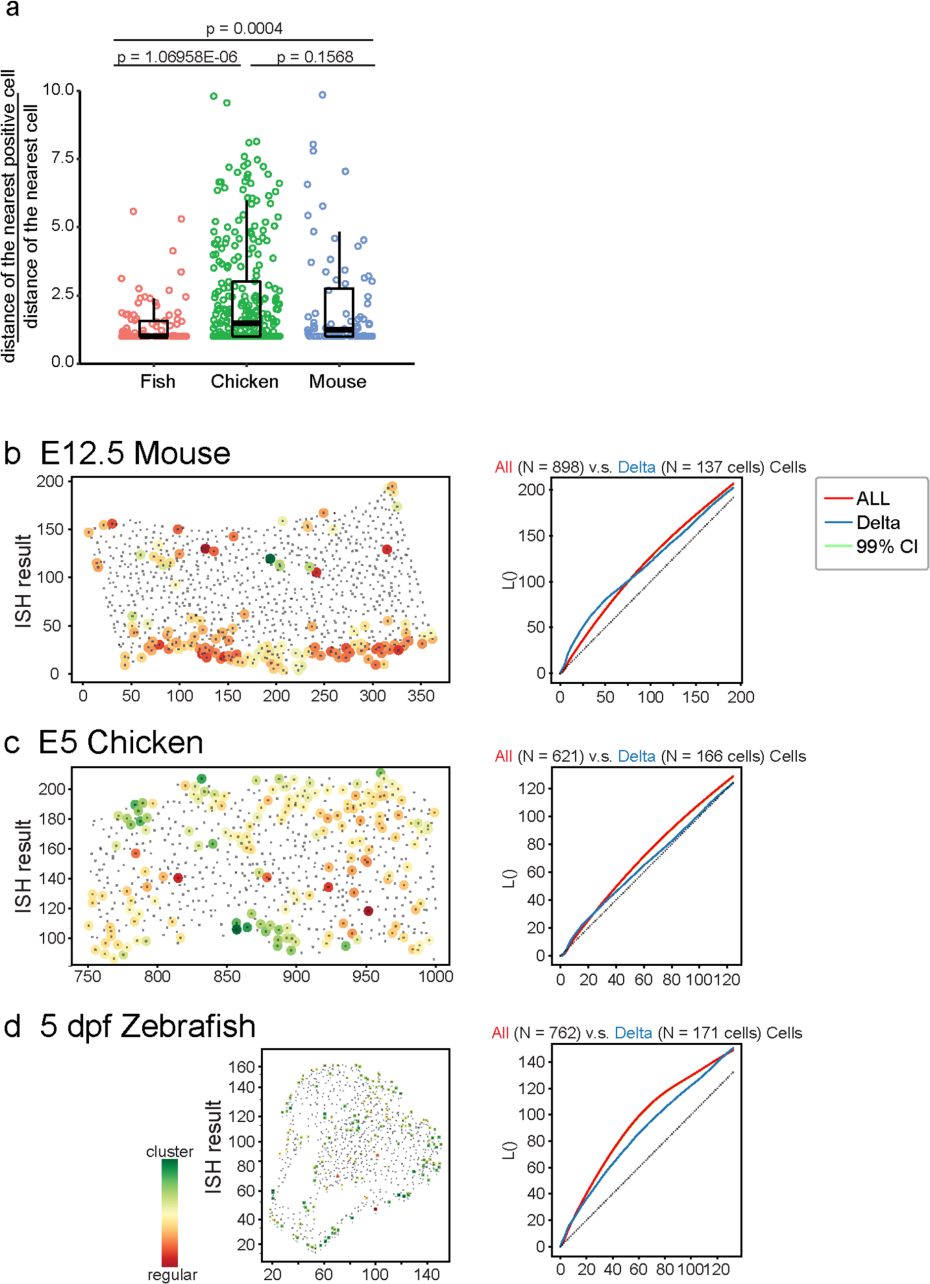
*dlc*-positive cells were stochastically distributed in the forebrain of larval zebrafish. **a** Quantitative analysis of the relative minimal distance between a signal-positive cell to the nearest signal-positive cell in the developing forebrain of 5 dpf larval zebrafishes, E5 chicken, and E12.5 mouse. * represent significance with *p* value < 0.01 by Student’s *t*-test. **b–d** L() analysis of the spatial distribution of *Dll1*-positive cells in the developing forebrain of E12.5 mouse (**d**), *Dll1*-positive cells in the developing forebrain of E5 chicken (**c**), and *dlc*-positive cells in the developing forebrain of 5 dpf larval zebrafish (**d**)

Previous reports have indicated that oscillated Notch signaling in mammalian neurogenesis is controlled by the homeostasis of oscillatory gene *Hes1*, which has an irregular salt-and-pepper pattern that differs from the simple conventional lateral inhibition with an ordered salt-and-pepper pattern [44, 45]. Therefore, in an oscillatory pattern where the distance between Hes1-positive cells varies, it is unlikely to fit the simple lateral inhibition situation where there is a great central tendency of distance between two signal-positive cells. To test this, we measured the minimal distance from *dlc/Dll1*-positive cells to the nearest *dlc/Dll1*-positive cells and the nearest cell (Fig. 3). Quantitative analysis showed that the pattern of minimal distance of *dlc*-positive cells in larval zebrafish was significantly different from *Dll1*-positive cells in both chickens and mice (Fig. 3a). If Dll1 expression was controlled solely by lateral inhibition, the coefficient of variation (c.v.) of the relative distance of the two nearest *Dll1*-positive cells would theoretically be limited. However, in line with the previous results for the developing mouse neocortex, the standard deviation of the relative distance was high and close to the average value, with c.v. = 0.8879 [12]. Similar to the results in mice, a moderate but not limited coefficient of variation for the relative distance of the two nearest *dlc*- or *Dll1*-positive cells was also observed in zebrafish forebrain at 5 dpf, with c.v. = 0.5803, and a high value for chicken cortex at embryonic day 5, with c.v. = 0.8121 (Fig. 3a). Notably, the relative distance between the two nearest *Dll1*-positive cells in zebrafish was significantly different compared to either chickens or mice, and no significance could be detected between chickens and mice.

If Notch signaling is not controlled solely by lateral inhibition, it may be activated periodically, specifically in an oscillatory pattern. To determine whether the distribution of *dlc/Dll1*-positive cells in the developing forebrain of zebrafish and chickens was similar to that in mice in which Dll1 was expressed in the known oscillatory pattern, we performed L function analysis, L(), to investigate the distribution of *dlc*/*Dll1*-positive cells in the germinal zone (Fig. 3b–d). Because the distribution of *dlc*/*Dll1*-positive cells was inherited from the distribution of all cells on the slices, the spatial patterns of *dlc*/*Dll1*-positive cells should be compared to the L() of all cells. L() analysis results showed that the distribution of *dlc*/*Dll1*-positive cells in the brains of all species analyzed in the present study was not random when compared to the simulated results but rather shared a similar distribution pattern of local regularity and global clustering (Fig. 3b–d and S6). This implies that the neural progenitors may be selected to express the Delta gene, *dlc/Dll1*, using a conserved mechanism across mice, chickens, and zebrafish.

### *dlc* Oscillates in the Developing Zebrafish Forebrain

As *dlc* oscillates during somite formation [23] and *dlc*-positive cells could be stochastically observed in the zebrafish forebrain (Figs. 2 and 3), we asked whether dlc was expressed in an oscillatory pattern during forebrain development, as has been observed in the developing mouse forebrain. To address this, we monitored the expression of dlc in the developing zebrafish forebrain using a transgenic fish, *tg(dlc::dlc-mCherry)*, in which normal somitogenesis was reported [34]. This transgenic strain contained 3 kbp 5′ upstream promoter region and 8 kbp full *dlc* sequence fused with mCherry/red fluorescent protein. To evaluate whether dlc-mCherry could serve as an appropriate reporter to monitor oscillatory expression, we examined the dynamic expression of dlc-mCherry in the PSM, where oscillatory dlc expression can be observed and is critical to somite formation. The live imaging results showed periodic mCherry expression and clear somite boundaries during 140 min of monitoring (Fig. S7a-c). Additional findings from crossing dlc-mCherry with *dlc* mutant zebrafish showed that somite defects could be partially rescued by dlc-mCherry, as shown by the observation of nine somites formed in dlc-mCherry crossed with dlc mutant zebrafish, compared to only four somites in dlc mutant zebrafish (Fig. S2a and S7d).

**Fig. 4:**
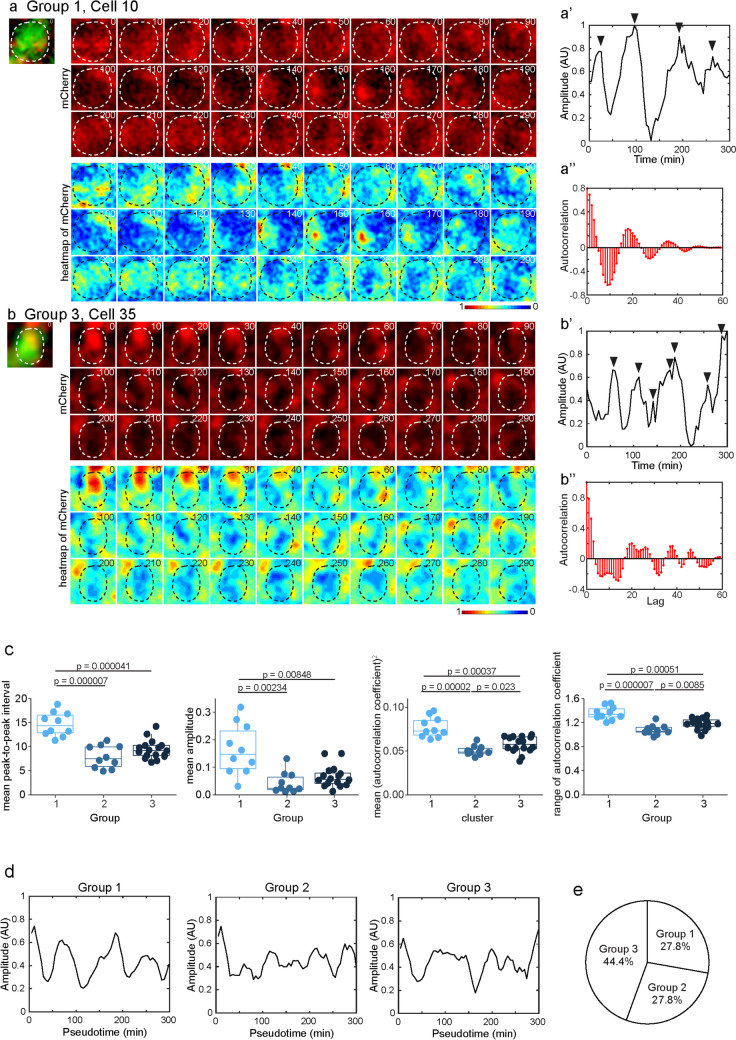
Fluctuating dlc expression was not synchronized in the developing forebrain of larval zebrafish. **a, b** Time-lapse imaging of mCherry signals and the heatmap of corrected mCherry signals in GFP cells (8*8 μm). Dashed lines delineate the cellular boundary of GFP cells. Time (minutes) was indicated. **a’, b’** Line charts show the amplitude of mCherry signals in cell 10 (**a’**) and cell 35 (**b’**). **a”, b”** Curve diagrams show the autocorrelation results of cell 10 (**a”**) and cell 35 (**b”**). **a, a’, a”** Time-lapse imaging and the analyses of cell 10 in group 1. **b, b’, b**” Time-lapse imaging and the analyses of cell 35 in group 3. **c** Box and dot plots show the mean peak-to-peak interval, mean amplitude, mean square of autocorrelation coefficient, and range of autocorrelation coefficient in three groups. *p* values by Student’s *t*-test are indicated. **d** Line charts show the mean amplitude in each group. **e** The pie chart shows the composition of analyzed GFP cells

The results of live imaging of mCherry signals and the rescue of somite defects by dlc-mCherry in *dlc* mutants suggested that the molecular behavior of dlc-mCherry is similar to innate dlc expression in controls. We then used this transgenic fish with dlc-mCherry to monitor dynamic dlc expression during forebrain development in zebrafish. A *GFP* construct was introduced into 4-cell-stage embryos via microinjection to label the shape of cells, and samples were collected at 2 dpf for 5 h of live-imaging recording (Fig. 4). To monitor dlc-mCherry signals within cells, we delineated cellular boundaries based on GFP expression and monitored the dynamic mCherry signals over a 300-min period (Fig. 4a–b”). The raw signals of mCherry were then processed, and peaks were determined using the imaging processing procedure described in “Materials and Methods,” and the resulting waves displaying the corrected mean mCherry signal intensity within individual cells were presented (Fig. 4a–b”, S8 and S9). In order to examine whether the waves matched the periodic pattern with a consistent amplitude and duration of period, we aligned the first peak of all analyzed cells as the zero of pseudo-timelines and applied autocorrelation analysis, a function to identify periodic patterns on MATLAB software (Fig. 4a”, b”, S8 and S9). Based on the results of the autocorrelation analysis (Fig. S9), we found that the dynamic expression patterns of dlc-mCherry were various and not apparently synchronized across all the cells tracked. Further clustering analysis based on the *k-means* machine learning strategy [46], three groups of oscillating behaviors could be clustered using 5 oscillating properties of robustness, including mean peak-to-peak interval, mean amplitude, mean square of autocorrelation coefficient, range of autocorrelation coefficient, and the number of cycles (Fig. 4c).

Among these three groups, group 1 exhibited a significant difference from groups 2 and 3 in terms of oscillating properties and the wave of mean amplitude (Fig. 4c, d). As shown in the montage of sequential images, the intensity of dlc-mCherry signals of cell 10 in group 1 displayed a clear up-and-down dynamic expression pattern, in contrast to that of cell 35 in group 3 (Fig. 4a–a’ and b–b’). Also, the wave of mean amplitude in each group showed a similar pattern as that of the individual cells (Fig. 4d and S8). The autocorrelation analysis of cells in group 1 showed distinct cycles between peaks and troughs, while cells in group 2 or 3 showed some connected peaks or troughs (Fig. 4a”, b” and S9). Collectively, our findings suggested that group 1 exhibited the most robust periodic behavior, whereas some cells in the other two groups also displayed periodic yet noisy waves (Fig. S8 and S9), indicating a distinct oscillatory pattern from the oscillatory Dll1 pattern in mice [12]. These results suggested the dlc oscillations of zebrafish forebrain progenitors occurred in a spectrum manner during determination (Fig. 4e), implying a prototypical pattern of dlc-involved Notch signaling in forebrain progenitors of zebrafish.

### Different Effects of Attenuating Notch Signaling on Neuronal Differentiation in Developing Forebrain of Zebrafish Compared to Chickens and Mice

If some of the dlc expression in the developing zebrafish forebrain exhibited an oscillatory pattern similar to that of Dll1 in the developing mouse forebrain, we then investigated the effects of attenuating this dlc-involved Notch signaling on neural differentiation during forebrain development in mice, chickens, and zebrafish. To this, we applied a γ-secretase inhibitor, MK-0752, to block Notch signaling during the critical period of neural differentiation. Several previous studies have demonstrated that blocking Notch signaling promotes neural differentiation in the cortical progenitors in the developing mouse cerebral cortex [6, 47]. Therefore, we first examined the effect of blocking Notch signaling using MK-0752 in mice.

**Fig. 5:**
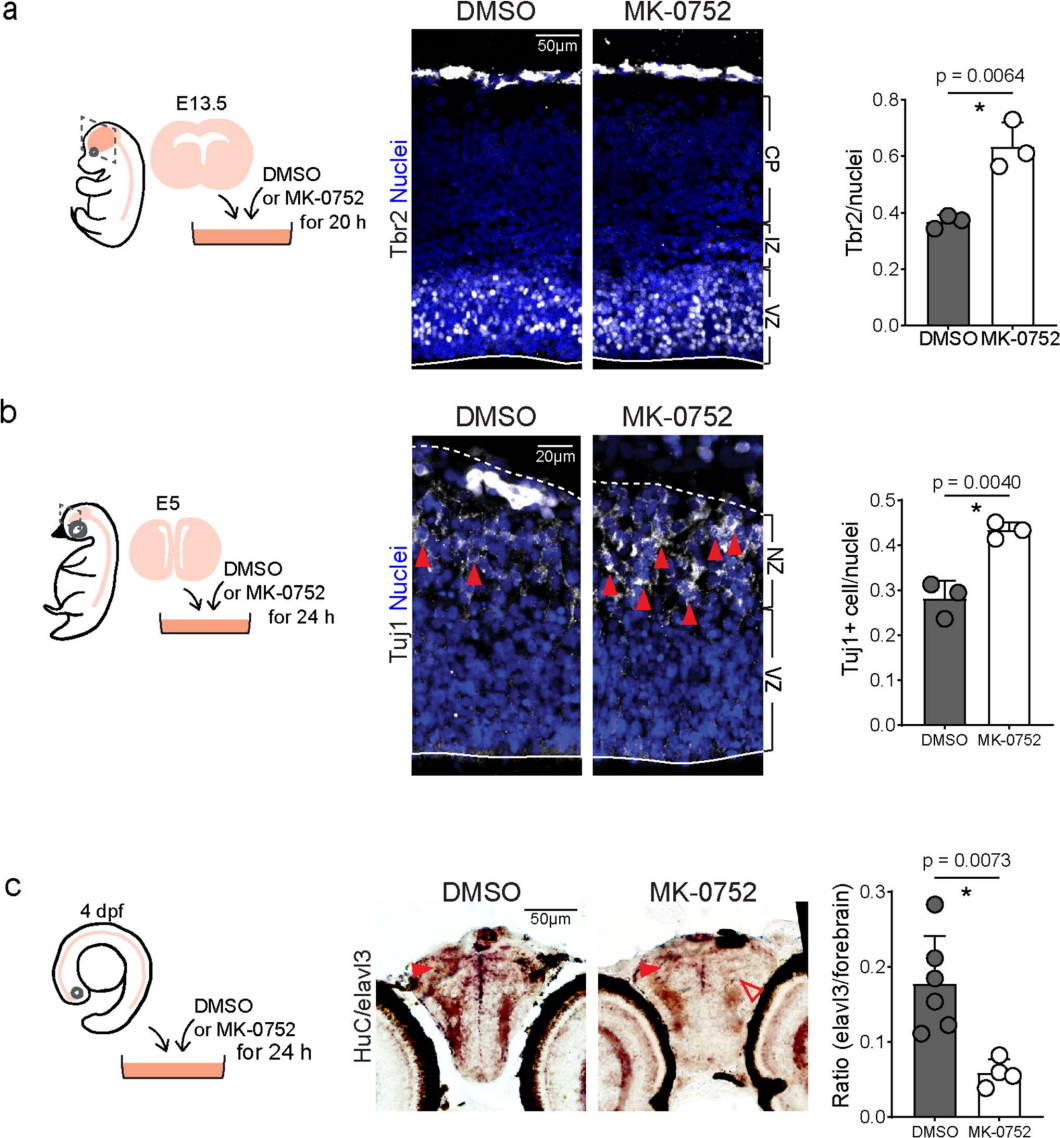
Blocking Notch signaling increased neural precursor numbers in developing mouse and chicken dorsal telencephalon but not in the larval zebrafish forebrain. **a** Schematic diagram of experimental design of MK-0752 treatment in developing mouse cerebral cortex, immunostaining and quantitative analysis of Tbr2-positive cells (nuclei) in VZ. VZ, ventricular zone; IZ, intermediate zone; CP, cortical plate. Solid lines indicate ventricular surface. Nuclei: DAPI staining. **b** Schematic diagram of experimental design of MK-0752 treatment in developing chicken pallium, immunostaining, and quantitative analysis of Tuj1-positive cells (nuclei) in NZ. Tuj1-positive cells are defined as nuclei surrounded by Tuj1-positive signals (arrowhead). Dashed lines indicate the pia surface and solid lines indicate the ventricular surface. Nuclei: DAPI staining. **c** Schematic diagram of experimental design of MK-0752 treatment in developing larval zebrafish forebrain, in situ hybridization, and quantitative analysis of area with *HuC/elavl3* signals. Close arrowheads indicate positive signals and open arrowheads indicate negative signals. Scale bars are indicated. Error bars represent standard deviation, and data points are shown in dots. *Significance with *p* value < 0.01 by Student’s *t*-test

Brain slice culture was utilized to prolong the exposure time to MK-0752 in the developing forebrain (Fig. 5a, left panel). As the Tbr2-positive intermediate progenitor population was primarily affected by the Notch signaling dysfunction condition [6], we measured Tbr2-positive intermediate progenitors after MK-0752 treatment. After 20 h of treatment, Tbr2-positive intermediate progenitors in the ventricular zone (VZ) were significantly increased in the MK-0752 treatment group compared to the DMSO control group, which is consistent with previous studies (Fig. 5a) [6, 47]. We then employed a similar slice culture procedure using chicken brain at E5 (Hamburger and Hamilton stages 24/25), administering MK-0752 or DMSO as the control in the culture medium for 24 h, during which the time chicken cortex experiences massive neurogenesis (Fig. 5b, left panel) [32]. As cortical progenitors in chicken and zebrafish undergo neural differentiation to produce neurons directly without producing Tbr2-positive intermediate progenitors [48], we measured the changes in neuron population in chicken and zebrafish brains after MK-0752 treatment. The immunostaining results showed that the number of Tuj1-positive neurons that expressed young neuronal gene Tuj1 was significantly increased in the neuronal zone (NZ) outside of the ventricular zone on chicken pallium (Fig. 5b), indicating that inhibiting Notch signaling with MK-0752 promotes neural differentiation in both chicken pallium and mouse cerebral cortex.

Finally, we investigated the effects of blocking Notch signaling in developing zebrafish forebrain. To avoid potential impact on somitogenesis, treating the zebrafish with MK-0752 from the fertilized egg stage is not applicable. Also, due to the challenge of specifically introducing MK-0752 to the zebrafish brain, we could not apply the method we used in mice and chickens. Thus, we treated larval zebrafish with MK-0752 at 4 dpf at 28.5 °C and assessed the effects on neuronal differentiation using in situ hybridization of the neural gene HuC/elavl3 in the forebrain 24 h after treatment (Fig. 5c, left panel). Surprisingly, we found a downregulation of neural gene *HuC/elavl3* in zebrafish forebrain after MK-0752 treatment, which contradicts our findings in mice and chickens (Fig. 5a, b) and in dlc mutant fish (Fig. 1g, h). This unexpected result, in conjunction with some oscillatory expression patterns of dlc in zebrafish, suggests that Notch signaling is rudimentary in the developing zebrafish forebrain when compared to the developing mouse neocortex.

## Discussion

The oscillatory expression pattern of Delta and Her is one of the most critical signatures of Notch signaling in regulating the differentiation of a progenitor population, such as vertebral somitogenesis and mammalian cortical development [10, 19]. One current hypothesis is that the biological function of the oscillatory pattern is to set up the preparation for rigorously controlled differentiation while the on-and-off Delta expression selects the cells to differentiate at the correct time point. This close-fitting oscillatory pattern, including regular amplitude and period, theoretically should be established during evolution. For the first time, undulating DeltaC/dlc, the functional ortholog of mammalian Dll1, expression was detected in the forebrain of larval zebrafish, while neither amplitude nor period was regular within and among cells. In addition to the undulation pattern being dissimilar to the typical one in mouse neocortical progenitors [12], the nearest dlc-oscillating cells were in the minority among the population, and the distance between *dlc*-positive cells of the forebrain of larval zebrafish was significantly different compared to chicken and mouse. Despite these inconsistencies, the distribution of *Dll1/dlc*-positive cells based on the results of L-function first showed clustering and then a regular distribution pattern in all three tested animals. Taken together, these data suggest that undulating dlc expression in the forebrain of larval zebrafish might be the prototype for the oscillatory pattern in mammalian neocortical progenitors [12, 49], and additional modulators should be acquired evolutionarily to establish the fully-fledged oscillatory pattern.

The rigorous regulatory machinery underlying oscillation was evolved for specific lineages, such as somite and forebrain. In our analysis using developing forebrains from multiple vertebrates, the spatial expression patterns of Delta and Her in neural progenitors were never consistent or even similar to those in developing somite [23, 50]. That might be due to the use of paralogs, upstream regulators, or downstream pathways. Taking mouse embryogenesis as an example, Hes1 oscillates in neocortical progenitors to regulate corticogenesis by inhibiting proneuronal gene *Ngn2* [12], and *Hes7* oscillates in the presomitic mesoderm to control somite formation by inhibiting glycosyltransferase gene *Lfng* [51]. Cyclic *Hes7* expression is modulated by Fgf and Wnt [52–54]. The variable components among lineages might be determined during evolution, which means that even the same lineage in different species may not employ the sequence-conserved ortholog.

In our study, we found that despite the increased expression of *dla* and *dlb* in the forebrains of *dlc* mutant fish, the phenotype of premature neurogenesis was not rescued, suggesting that in zebrafish, dlc instead of dla, dld, or dld is utilized in the pallial progenitors of developing forebrain. dlc was specifically expressed in forebrain progenitors and oscillated in some of them, while dld was not specifically enriched in neural progenitors. Despite the conserved dynamics and spatial expression patterns of dlc and Dll1, the reason why mammals utilize a sequence non-conserved *Delta* gene to regulate forebrain development remains obscure, especially when the DSL domain is present in Dll1 but absent in dlc [55]. It is also uncertain whether the Dll1-sequence-conserved dld, with its high expression pattern in the forebrain, plays a role in forebrain development. Considering the function of the DSL domain in mediating Notch signaling, it is possible that the incorporation of DSL might contribute to establishing the mature Notch signaling pattern to create the functional neocortex in mammals.

The period of oscillatory Her expression was controlled by the presence of Notch signaling [54]. Blocking Notch signaling via γ-secretase inhibitor affected the oscillatory pattern, downregulated Her expression, and induced differentiation of germinal cells, such as in neural tube and presomitic mesoderm [56]. In this scheme, differentiated cells massively increased after treatment [57]. However, neuron numbers were significantly reduced in the developing forebrains of MK-0752-treated zebrafish, which was inconsistent with the forebrains of MK-0752-treated chickens and mice. γ-Secretase inhibitors have been utilized to block Notch signaling in various tissues, including PSM [58] and central nervous system [12, 59], across vertebrate species. Although MK-0752 has been reported as a specific γ-secretase inhibitor [60] and γ-secretase has proteolytic targets other than Notch1 [61], such as APP (amyloid precursor protein), E-cadherin/Cdh1, ErbB4, CD44, tyrosinase, TREM2, and Alcadein/Alcs, the ISH results of mouse developing neocortex during cortical expansion period, E11.5, using Allen Brain Atlas (https://portal.brain-map.org) and previous reports [62–64] suggested Notch1 is the only one gene generally expressed in the developing neocortex (Fig. S10) and Notch signaling was most affected signaling pathway in early development [65]. Thus, the inconsistency found in zebrafish was most likely due to either the immature oscillatory pattern or inadequate oscillatory cells in the developing zebrafish forebrain. In support of this, the inconsistent changes in the progenitor pool in *dlc* mutant zebrafish versus conditional *Dll1* mutant neocortex suggest that the process of stem cells generating neural progenitors may also be regulated by Notch signaling, and this switch from symmetric to asymmetric division has already finished [40]. Taken together, our data demonstrate that sophisticated control of stepwise neural differentiation with accurate timing and at the precise level is critical to establishing a functional neocortex for complex sensory processing.

## Conclusions

Notch signaling is involved in the development of multiple lineages, with Delta/Notch and the effector Her/Hes acting as protagonists to modulate the developmental clocks and control downstream pathways. While deficiencies in Notch signaling cause severe brain malformation in mammals, *dlc* mutant fish survive with intact forebrain structure, suggesting alterations in Notch signaling during forebrain evolution. In this study, for the first time, we revealed the functional homolog of Dll1, dlc, has a similar expression pattern in the developing forebrain as in mammals and oscillates in a subset of neural progenitors of the zebrafish forebrain, similar to the Dll1 expression pattern in the mammalian neocortical progenitors. Moreover, blocking the dlc-involved Notch signaling pathway by a γ-secretase inhibitor impaired neuronal differentiation in the developing zebrafish forebrain, contradicting our findings in mice and chickens and in dlc mutant fish. Taken together, our results demonstrate the prototype of Notch signaling mediated by dlc in regulating neurogenesis of the developing zebrafish forebrain, which may serve as a basis for establishing the pattern of the complex mammalian neocortex.

## Supplementary Information

Below is the link to the electronic supplementary material.

Supplementary file1 Fig. S1 Phylogenic tree of Delta family among vertebrates; Fig. S2 Affected somite formation in dlc mutant embryos and the nuclei staining in zebrafish forebrain; Fig. S3 Increased neurons and progenitors in the posterior forebrain of dlc mutant larval zebrafish; Fig. S4 The expression of delta genes and the effector gene of Notch signaling in dlc mutant fish; Fig. S5 dld and her3 expression in the forebrain of larval zebrafish at 5 dpf; Fig. S6 The simulation of clustering, regularity, and randomness pattern of Delta/Dll1-positive cells in mice, chickens, and zebrafish developing pallium; Fig. S7 PSM live imaging of dlc-mCherry transgenic fish showing rescued oscillatory expression during early somitogenesis; Fig. S8 Dynamic dlc-mCherry signals in the dorsal telencephalon cells over a 300-minute period; Fig. S9 Autocorrelation results of corrected dlc-mCherry signals in the dorsal telencephalon cells over a 300-minute period. Fig. S10 Expression pattern of the targets of γ-secretase in the developing neocortex at E11.5; Fig. S11 The procedure of imaging processing for ISH results; Table S1 Primers for ISH probe preparation. (PDF 19434 KB)

## Data Availability

The data that support the findings of this study are available from the corresponding author upon reasonable request.

## References

[1] Werner JM, Negesse MY, Brooks DL, Caldwell AR, Johnson JM, Brewster RM (2021) Hallmarks of primary neurulation are conserved in the zebrafish forebrain. Commun Biol 4(1):147. 10.1038/s42003-021-01655-8 33514864 10.1038/s42003-021-01655-8 PMC7846805

[2] Chen CC, Winkler CM, Pfenning AR, Jarvis ED (2013) Molecular profiling of the developing avian telencephalon: regional timing and brain subdivision continuities. J Comp Neurol 521(16):3666–3701. 10.1002/cne.23406 23818174 10.1002/cne.23406 PMC3863995

[3] Reillo I, de Juan RC, Garcia-Cabezas MA, Borrell V (2011) A role for intermediate radial glia in the tangential expansion of the mammalian cerebral cortex. Cereb Cortex 21(7):1674–1694. 10.1093/cercor/bhq238 21127018 10.1093/cercor/bhq238

[4] Mione M, Shanmugalingam S, Kimelman D, Griffin K (2001) Overlapping expression of zebrafish T-brain-1 and eomesodermin during forebrain development. Mech Dev 100(1):93–97. 10.1016/s0925-4773(00)00501-3 11118891 10.1016/s0925-4773(00)00501-3

[5] Gaiano N, Nye JS, Fishell G (2000) Radial glial identity is promoted by Notch1 signaling in the murine forebrain. Neuron 26(2):395–404 10839358 10.1016/s0896-6273(00)81172-1

[6] Mizutani K-i, Yoon K, Dang L, Tokunaga A, Gaiano N (2007) Differential Notch signalling distinguishes neural stem cells from intermediate progenitors. Nature 449(7160):351–355 17721509 10.1038/nature06090

[7] Kageyama R, Ohtsuka T (1999) The Notch-Hes pathway in mammalian neural development. Cell Res 9(3):179–188. 10.1038/sj.cr.7290016 10520600 10.1038/sj.cr.7290016

[8] Sjoqvist M, Andersson ER (2019) Do as I say, Not(ch) as I do: lateral control of cell fate. Dev Biol 447(1):58–70. 10.1016/j.ydbio.2017.09.032 28969930 10.1016/j.ydbio.2017.09.032

[9] Pan D, Rubin GM (1997) Kuzbanian controls proteolytic processing of Notch and mediates lateral inhibition during Drosophila and vertebrate neurogenesis. Cell 90(2):271–280. 10.1016/s0092-8674(00)80335-9 9244301 10.1016/s0092-8674(00)80335-9

[10] Jiang YJ, Aerne BL, Smithers L, Haddon C, Ish-Horowicz D, Lewis J (2000) Notch signalling and the synchronization of the somite segmentation clock. Nature 408(6811):475–479. 10.1038/35044091 11100729 10.1038/35044091

[11] Kageyama R, Ohtsuka T, Shimojo H, Imayoshi I (2008) Dynamic Notch signaling in neural progenitor cells and a revised view of lateral inhibition. Nat Neurosci 11(11):1247–1251. 10.1038/nn.2208 18956012 10.1038/nn.2208

[12] Shimojo H, Ohtsuka T, Kageyama R (2008) Oscillations in notch signaling regulate maintenance of neural progenitors. Neuron 58(1):52–64. 10.1016/j.neuron.2008.02.014 18400163 10.1016/j.neuron.2008.02.014

[13] Mueller T, Wullimann MF (2016) Chapter 2 - Atlas of cellular markers in zebrafish neurogenesis: Atlas. In: Mueller T, Wullimann MF (eds) Atlas of Early Zebrafish Brain Development, 2nd Edn. Elsevier, San Diego, pp 27–157. 10.1016/B978-0-12-418669-9.00002-7

[14] Alunni A, Bally-Cuif L (2016) A comparative view of regenerative neurogenesis in vertebrates. Development 143(5):741–753. 10.1242/dev.122796 26932669 10.1242/dev.122796 PMC4813331

[15] Zambusi A, Ninkovic J (2020) Regeneration of the central nervous system-principles from brain regeneration in adult zebrafish. World J Stem Cells 12(1):8–24. 10.4252/wjsc.v12.i1.8 32110272 10.4252/wjsc.v12.i1.8 PMC7031763

[16] Alunni A, Krecsmarik M, Bosco A, Galant S, Pan L, Moens CB, Bally-Cuif L (2013) Notch3 signaling gates cell cycle entry and limits neural stem cell amplification in the adult pallium. Development 140(16):3335–3347. 10.1242/dev.095018 23863484 10.1242/dev.095018 PMC3737716

[17] Diotel N, Lübke L, Strähle U, Rastegar S (2020) Common and distinct features of adult neurogenesis and regeneration in the telencephalon of zebrafish and mammals. Front Neurosci 14:568930. 10.3389/fnins.2020.568930 33071740 10.3389/fnins.2020.568930 PMC7538694

[18] Sueda R, Imayoshi I, Harima Y, Kageyama R (2019) High Hes1 expression and resultant Ascl1 suppression regulate quiescent vs. active neural stem cells in the adult mouse brain. Genes Dev 33(9–10):511–523. 10.1101/gad.323196.118 30862661 10.1101/gad.323196.118 PMC6499325

[19] Ohtsuka T, Ishibashi M, Gradwohl G, Nakanishi S, Guillemot F, Kageyama R (1999) Hes1 and Hes5 as notch effectors in mammalian neuronal differentiation. EMBO J 18(8):2196–2207. 10.1093/emboj/18.8.2196 10205173 10.1093/emboj/18.8.2196 PMC1171303

[20] Ishibashi M, Ang SL, Shiota K, Nakanishi S, Kageyama R, Guillemot F (1995) Targeted disruption of mammalian hairy and Enhancer of split homolog-1 (HES-1) leads to up-regulation of neural helix-loop-helix factors, premature neurogenesis, and severe neural tube defects. Genes Dev 9(24):3136–3148. 10.1101/gad.9.24.3136 8543157 10.1101/gad.9.24.3136

[21] Fischer-Zirnsak B, Segebrecht L, Schubach M, Charles P, Alderman E, Brown K, Cadieux-Dion M, Cartwright T et al (2019) Haploinsufficiency of the Notch ligand DLL1 causes variable neurodevelopmental disorders. Am J Hum Genet 105(3):631–639. 10.1016/j.ajhg.2019.07.002 31353024 10.1016/j.ajhg.2019.07.002 PMC6731356

[22] Tokita M, Kuratani S (2001) Normal embryonic stages of the Chinese softshelled turtle Pelodiscus sinensis (Trionychidae). Zoolog Sci 18(5):705–715. 10.2108/zsj.18.705

[23] Julich D, Hwee Lim C, Round J, Nicolaije C, Schroeder J, Davies A, Geisler R, Lewis J et al (2005) beamter/deltaC and the role of Notch ligands in the zebrafish somite segmentation, hindbrain neurogenesis and hypochord differentiation. Dev Biol 286(2):391–404. 10.1016/j.ydbio.2005.06.040 16125692 10.1016/j.ydbio.2005.06.040

[24] Hou PS, Kumamoto T, Hanashima C (2017) A sensitive and versatile in situ hybridization protocol for gene expression analysis in developing amniote brains. Methods Mol Biol 1650:319–334. 10.1007/978-1-4939-7216-6_22 28809032 10.1007/978-1-4939-7216-6_22

[25] Hou PS, Miyoshi G, Hanashima C (2019) Sensory cortex wiring requires preselection of short- and long-range projection neurons through an Egr-Foxg1-COUP-TFI network. Nat Commun 10(1):3581. 10.1038/s41467-019-11043-w 31395862 10.1038/s41467-019-11043-w PMC6687716

[26] Schindelin J, Arganda-Carreras I, Frise E, Kaynig V, Longair M, Pietzsch T, Preibisch S, Rueden C et al (2012) Fiji: an open-source platform for biological-image analysis. Nat Methods 9(7):676–682. 10.1038/nmeth.2019 22743772 10.1038/nmeth.2019 PMC3855844

[27] Schroeder AB, Dobson ETA, Rueden CT, Tomancak P, Jug F, Eliceiri KW (2021) The ImageJ ecosystem: open-source software for image visualization, processing, and analysis. Protein Sci 30(1):234–249. 10.1002/pro.3993 33166005 10.1002/pro.3993 PMC7737784

[28] Bell ML, Earl JB, Britt SG (2007) Two types of Drosophila R7 photoreceptor cells are arranged randomly: a model for stochastic cell-fate determination. J Comp Neurol 502(1):75–85. 10.1002/cne.21298 17335038 10.1002/cne.21298

[29] Lagache T, Lang G, Sauvonnet N, Olivo-Marin JC (2013) Analysis of the spatial organization of molecules with robust statistics. PLoS ONE 8(12):e80914. 10.1371/journal.pone.0080914 24349021 10.1371/journal.pone.0080914 PMC3857798

[30] Tsai MH, Cheng HY, Nian FS, Liu C, Chao NH, Chiang KL, Chen SF, Tsai JW (2020) Impairment in dynein-mediated nuclear translocation by BICD2 C-terminal truncation leads to neuronal migration defect and human brain malformation. Acta Neuropathol Commun 8(1):106. 10.1186/s40478-020-00971-0 32665036 10.1186/s40478-020-00971-0 PMC7362644

[31] Yang C, Li X, Li S, Chai X, Guan L, Qiao L, Li H, Lin J (2019) Organotypic slice culture based on in ovo electroporation for chicken embryonic central nervous system. J Cell Mol Med 23(3):1813–1826. 10.1111/jcmm.14080 30565384 10.1111/jcmm.14080 PMC6378233

[32] Nomura T, Nagao K, Shirai R, Gotoh H, Umeda M, Ono K (2022) Temperature sensitivity of Notch signaling underlies species-specific developmental plasticity and robustness in amniote brains. Nat Commun 13(1):96. 10.1038/s41467-021-27707-5 35013223 10.1038/s41467-021-27707-5 PMC8748702

[33] Harbor CS (2011) E3 medium (for zebrafish embryos). Cold Spring Harb Protoc. 10.1101/pdb.rec066449

[34] Chen Y-C, Liao B-K, Lu Y-F, Liu Y-H, Hsieh F-C, Hwang P-P, Hwang S-PL (2019) Zebrafish Klf4 maintains the ionocyte progenitor population by regulating epidermal stem cell proliferation and lateral inhibition. PLoS Genet 15(4):e1008058. 10.1371/journal.pgen.1008058 30933982 10.1371/journal.pgen.1008058 PMC6459544

[35] Yoshioka-Kobayashi K, Matsumiya M, Niino Y, Isomura A, Kori H, Miyawaki A, Kageyama R (2020) Coupling delay controls synchronized oscillation in the segmentation clock. Nature 580(7801):119–123. 10.1038/s41586-019-1882-z 31915376 10.1038/s41586-019-1882-z

[36] Liao BK, Jorg DJ, Oates AC (2016) Faster embryonic segmentation through elevated Delta-Notch signalling. Nat Commun 7:11861. 10.1038/ncomms11861 27302627 10.1038/ncomms11861 PMC4912627

[37] Oates AC, Ho RK (2002) Hairy/E(spl)-related (Her) genes are central components of the segmentation oscillator and display redundancy with the Delta/Notch signaling pathway in the formation of anterior segmental boundaries in the zebrafish. Development 129(12):2929–2946. 10.1242/dev.129.12.2929 12050140 10.1242/dev.129.12.2929

[38] Lein ES, Hawrylycz MJ, Ao N, Ayres M, Bensinger A, Bernard A, Boe AF, Boguski MS et al (2007) Genome-wide atlas of gene expression in the adult mouse brain. Nature 445(7124):168–176. 10.1038/nature05453 17151600 10.1038/nature05453

[39] Kroehne V, Freudenreich D, Hans S, Kaslin J, Brand M (2011) Regeneration of the adult zebrafish brain from neurogenic radial glia-type progenitors. Development 138(22):4831–4841. 10.1242/dev.072587 22007133 10.1242/dev.072587

[40] Kawaguchi D, Yoshimatsu T, Hozumi K, Gotoh Y (2008) Selection of differentiating cells by different levels of delta-like 1 among neural precursor cells in the developing mouse telencephalon. Development 135(23):3849–3858. 10.1242/dev.024570 18997111 10.1242/dev.024570

[41] Hall ZJ, Tropepe V (2018) Movement maintains forebrain neurogenesis via peripheral neural feedback in larval zebrafish. Elife 7. 10.7554/eLife.31045 10.7554/eLife.31045 PMC5847330 29528285

[42] Abdel-Mannan O, Cheung AF, Molnar Z (2008) Evolution of cortical neurogenesis. Brain Res Bull 75(2–4):398–404. 10.1016/j.brainresbull.2007.10.047 18331905 10.1016/j.brainresbull.2007.10.047

[43] Nelson BR, Hodge RD, Bedogni F, Hevner RF (2013) Dynamic interactions between intermediate neurogenic progenitors and radial glia in embryonic mouse neocortex: potential role in Dll1-Notch signaling. J Neurosci 33(21):9122–9139 23699523 10.1523/JNEUROSCI.0791-13.2013 PMC3716275

[44] Ochi S, Imaizumi Y, Shimojo H, Miyachi H, Kageyama R (2020) Oscillatory expression of Hes1 regulates cell proliferation and neuronal differentiation in the embryonic brain. Development 147(4). 10.1242/dev.182204 10.1242/dev.182204 32094111

[45] Matsuda M, Hayashi H, Garcia-Ojalvo J, Yoshioka-Kobayashi K, Kageyama R, Yamanaka Y, Ikeya M, Toguchida J et al (2020) Species-specific segmentation clock periods are due to differential biochemical reaction speeds. Science 369(6510):1450–1455. 10.1126/science.aba7668 32943519 10.1126/science.aba7668

[46] Maharaj EA, D’Urso P, Caiado J (2019) Time series clustering and classification. CRC Press

[47] Kawaguchi A, Ikawa T, Kasukawa T, Ueda H, Kurimoto K, Saitou M, Matsuzaki F (2008) Single-cell gene profiling defines differential progenitor subclasses in mammalian neurogenesis. Development (Cambridge, England) 135:3113–3124. 10.1242/dev.022616 18725516 10.1242/dev.022616

[48] Cardenas A, Borrell V (2020) Molecular and cellular evolution of corticogenesis in amniotes. Cell Mol Life Sci 77(8):1435–1460. 10.1007/s00018-019-03315-x 31563997 10.1007/s00018-019-03315-x PMC11104948

[49] Trujillo CA, Gao R, Negraes PD, Gu J, Buchanan J, Preissl S, Wang A, Wu W et al (2019) Complex oscillatory waves emerging from cortical organoids model early human brain network development. Cell Stem Cell 25(4):558–569 e557. 10.1016/j.stem.2019.08.002 10.1016/j.stem.2019.08.002 PMC6778040 31474560

[50] Bessho Y, Sakata R, Komatsu S, Shiota K, Yamada S, Kageyama R (2001) Dynamic expression and essential functions of Hes7 in somite segmentation. Genes Dev 15(20):2642–2647. 10.1101/gad.930601 11641270 10.1101/gad.930601 PMC312810

[51] Bessho Y, Hirata H, Masamizu Y, Kageyama R (2003) Periodic repression by the bHLH factor Hes7 is an essential mechanism for the somite segmentation clock. Genes Dev 17(12):1451–1456. 10.1101/gad.1092303 12783854 10.1101/gad.1092303 PMC196074

[52] Naiche LA, Holder N, Lewandoski M (2011) FGF4 and FGF8 comprise the wavefront activity that controls somitogenesis. Proc Natl Acad Sci U S A 108(10):4018–4023. 10.1073/pnas.1007417108 21368122 10.1073/pnas.1007417108 PMC3054031

[53] Dunty WC Jr, Biris KK, Chalamalasetty RB, Taketo MM, Lewandoski M, Yamaguchi TP (2008) Wnt3a/beta-catenin signaling controls posterior body development by coordinating mesoderm formation and segmentation. Development 135(1):85–94. 10.1242/dev.009266 18045842 10.1242/dev.009266

[54] Morelli LG, Ares S, Herrgen L, Schroter C, Julicher F, Oates AC (2009) Delayed coupling theory of vertebrate segmentation. HFSP J 3(1):55–66. 10.2976/1.3027088 19492022 10.2976/1.3027088 PMC2689616

[55] Nian FS, Hou PS (2022) Evolving roles of Notch signaling in cortical development. Front Neurosci 16:844410. 10.3389/fnins.2022.844410 35422684 10.3389/fnins.2022.844410 PMC9001970

[56] Ozbudak EM, Lewis J (2008) Notch signalling synchronizes the zebrafish segmentation clock but is not needed to create somite boundaries. PLoS Genet 4(2):e15. 10.1371/journal.pgen.0040015 18248098 10.1371/journal.pgen.0040015 PMC2222926

[57] Crawford TQ, Roelink H (2007) The notch response inhibitor DAPT enhances neuronal differentiation in embryonic stem cell-derived embryoid bodies independently of sonic hedgehog signaling. Dev Dyn 236(3):886–892. 10.1002/dvdy.21083 17295317 10.1002/dvdy.21083

[58] Riedel-Kruse IH, Muller C, Oates AC (2007) Synchrony dynamics during initiation, failure, and rescue of the segmentation clock. Science 317(5846):1911–1915. 10.1126/science.1142538 17702912 10.1126/science.1142538

[59] Rothenaigner I, Krecsmarik M, Hayes JA, Bahn B, Lepier A, Fortin G, Gotz M, Jagasia R, Bally-Cuif L (2011) Clonal analysis by distinct viral vectors identifies bona fide neural stem cells in the adult zebrafish telencephalon and characterizes their division properties and fate. Development 138(8):1459–1469. 10.1242/dev.058156 21367818 10.1242/dev.058156

[60] Krop I, Demuth T, Guthrie T, Wen PY, Mason WP, Chinnaiyan P, Butowski N, Groves MD et al (2012) Phase I pharmacologic and pharmacodynamic study of the gamma secretase (Notch) inhibitor MK-0752 in adult patients with advanced solid tumors. J Clin Oncol 30(19):2307–2313. 10.1200/JCO.2011.39.1540 22547604 10.1200/JCO.2011.39.1540

[61] Zhang X, Li Y, Xu H, Zhang YW (2014) The gamma-secretase complex: from structure to function. Front Cell Neurosci 8:427. 10.3389/fncel.2014.00427 25565961 10.3389/fncel.2014.00427 PMC4263104

[62] Tief K, Schmidt A, Beermann F (1998) New evidence for presence of tyrosinase in substantia nigra, forebrain and midbrain. Brain Res Mol Brain Res 53(1–2):307–310. 10.1016/s0169-328x(97)00301-x 9473705 10.1016/s0169-328x(97)00301-x

[63] Jay TR, Hirsch AM, Broihier ML, Miller CM, Neilson LE, Ransohoff RM, Lamb BT, Landreth GE (2017) Disease progression-dependent effects of TREM2 deficiency in a mouse model of Alzheimer’s disease. J Neurosci 37(3):637–647. 10.1523/JNEUROSCI.2110-16.2016 28100745 10.1523/JNEUROSCI.2110-16.2016 PMC5242410

[64] Araki Y, Kawano T, Taru H, Saito Y, Wada S, Miyamoto K, Kobayashi H, Ishikawa HO et al (2007) The novel cargo Alcadein induces vesicle association of kinesin-1 motor components and activates axonal transport. EMBO J 26(6):1475–1486. 10.1038/sj.emboj.7601609 17332754 10.1038/sj.emboj.7601609 PMC1829376

[65] Geling A, Steiner H, Willem M, Bally-Cuif L, Haass C (2002) A gamma-secretase inhibitor blocks Notch signaling in vivo and causes a severe neurogenic phenotype in zebrafish. EMBO Rep 3(7):688–694. 10.1093/embo-reports/kvf124 12101103 10.1093/embo-reports/kvf124 PMC1084181

