# Supplementary material for: Oscillatory DeltaC Expression in Neural Progenitors Primes the Prototype of Forebrain Development
Source: Mol Neurobiol. 2024 Oct 11;62(4):4076–92. doi: 10.1007/s12035-024-04530-9 (PMC11880136; doi:10.1007/s12035-024-04530-9)
Supplement: Supplementary file 1 — Supplementary file1 Fig. S1 Phylogenic tree of Delta family among vertebrates; Fig. S2 Affected somite formation in dlc mutant embryos and the nuclei staining in zebrafish forebrain; Fig. S3 Increased neurons and progenitors in the posterior forebrain of dlc mutant larval zebrafish; Fig. S4 The expression of delta genes and the effector gene of Notch signaling in dlc mutant fish; Fig. S5 dld and her3 expression in the forebrain of larval zebrafish at 5 dpf; Fig. S6 The simulation of clustering, regularity, and randomness pattern of Delta/Dll1-positive cells in mice, chickens, and zebrafish developing pallium; Fig. S7 PSM live imaging of dlc-mCherry transgenic fish showing rescued oscillatory expression during early somitogenesis; Fig. S8 Dynamic dlc-mCherry signals in the dorsal telencephalon cells over a 300-minute period; Fig. S9 Autocorrelation results of corrected dlc-mCherry signals in the dorsal telencephalon cells over a 300-minute period. Fig. S10 Expression pattern of the targets of γ-secretase in the developing neocortex at E11.5; Fig. S11 The procedure of imaging processing for ISH results; Table S1 Primers for ISH probe preparation. (PDF 19434 KB) [file 12035_2024_4530_MOESM1_ESM.pdf]

Supplementary Information

Oscillatory DeltaC expression in neural progenitors primes the prototype of forebrain development

Fan-Shin Nian, Bo-Kai Liao, Yen-Lin Su, Pei-Rong Wu, Jin-Wu Tsai, Pei-Shan Hou

Fig. S1
Fig. S2
Fig. S3
Fig. S4
Fig. S5
Fig. S6
Fig. S7
Fig. S8
Fig. S9
Fig. S10
Fig. S11
Table S1

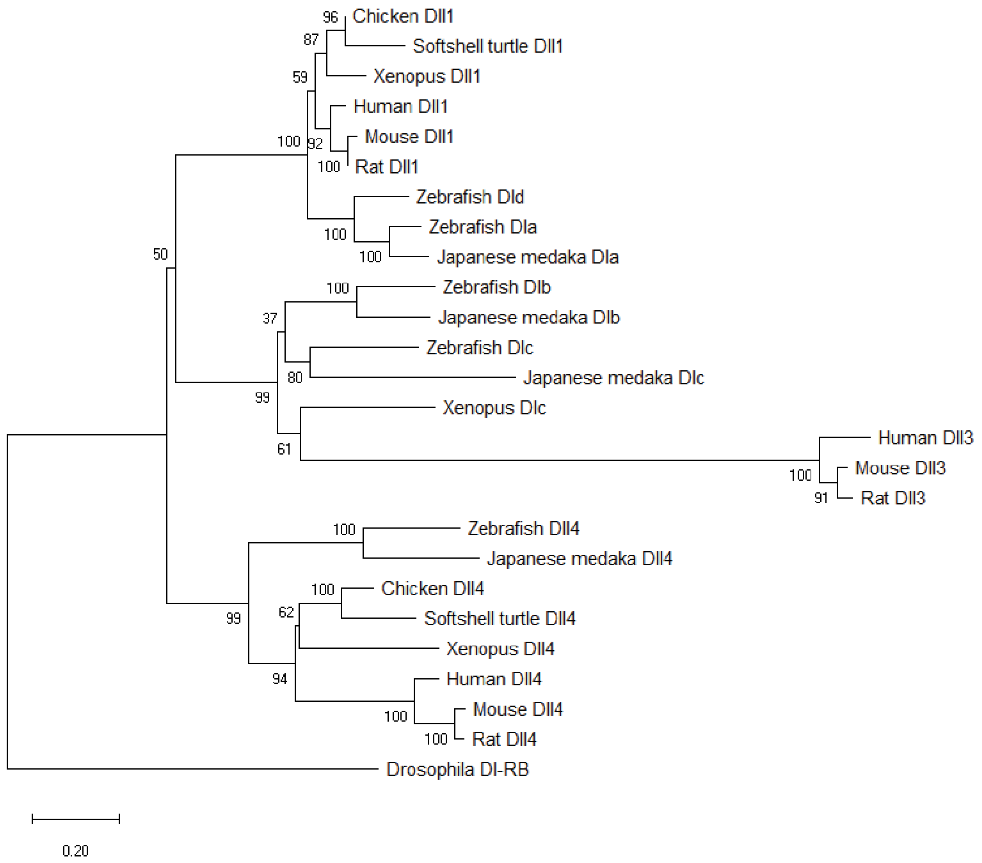

Fig. S1. Phylogenic tree of Delta family among vertebrates
The number at the nodes represents the percentage of bootstrap support. Scale bar: 0.2 nucleotide substitutions per site.

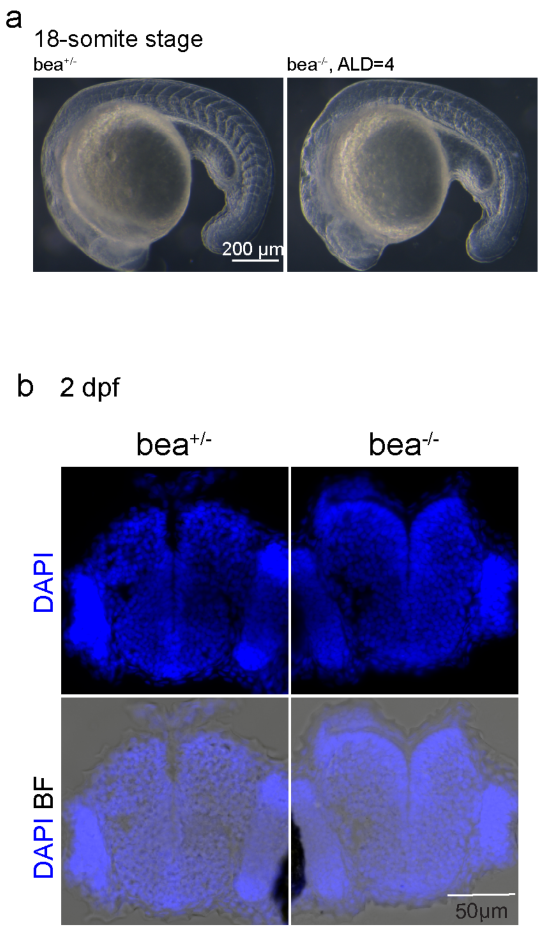

Fig. S2. Affected somite formation in *dlc* mutant embryos and the nuclei staining in zebrafish forebrain (a) Lateral view of *bea*$^{+/-}$ and *bea*$^{-/-}$ mutant embryos at 18-somite stage. ALD, anterior limited defect. (b) Nuclei staining in the forebrain of 2 dpf *bea*$^{+/-}$ and *bea*$^{-/-}$ zebrafish. Scale bars are indicated. BF: bright field image.

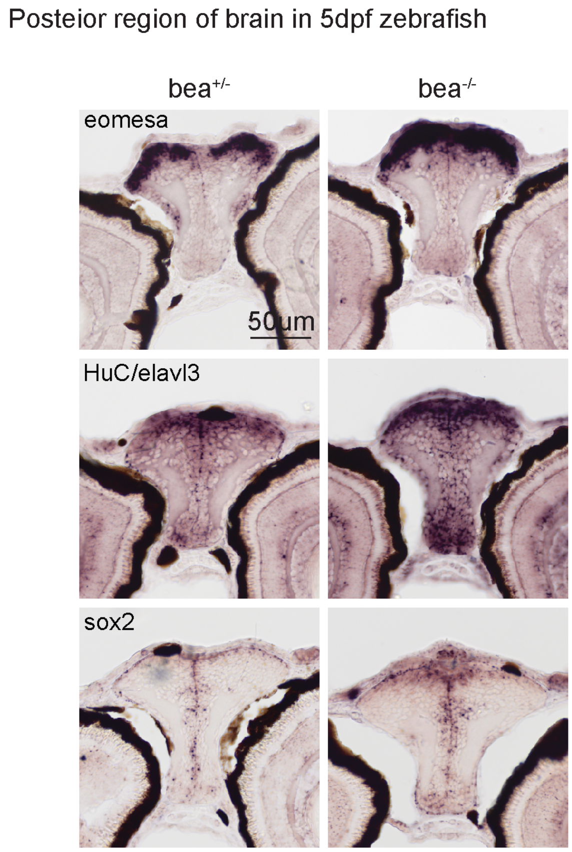

Fig. S3. Increased neurons and progenitors in the posterior forebrain of *dlc* mutant larval zebrafish In situ hybridization of larval zebrafish at 5 dpf using probes specifically targeting progenitor gene *sox2*, neuronal precursor gene *eomesa*, and neuronal gene *HuC/elavl3*. Scale bars are indicated. The result was from the posterior region of the same brains in Fig. 1.

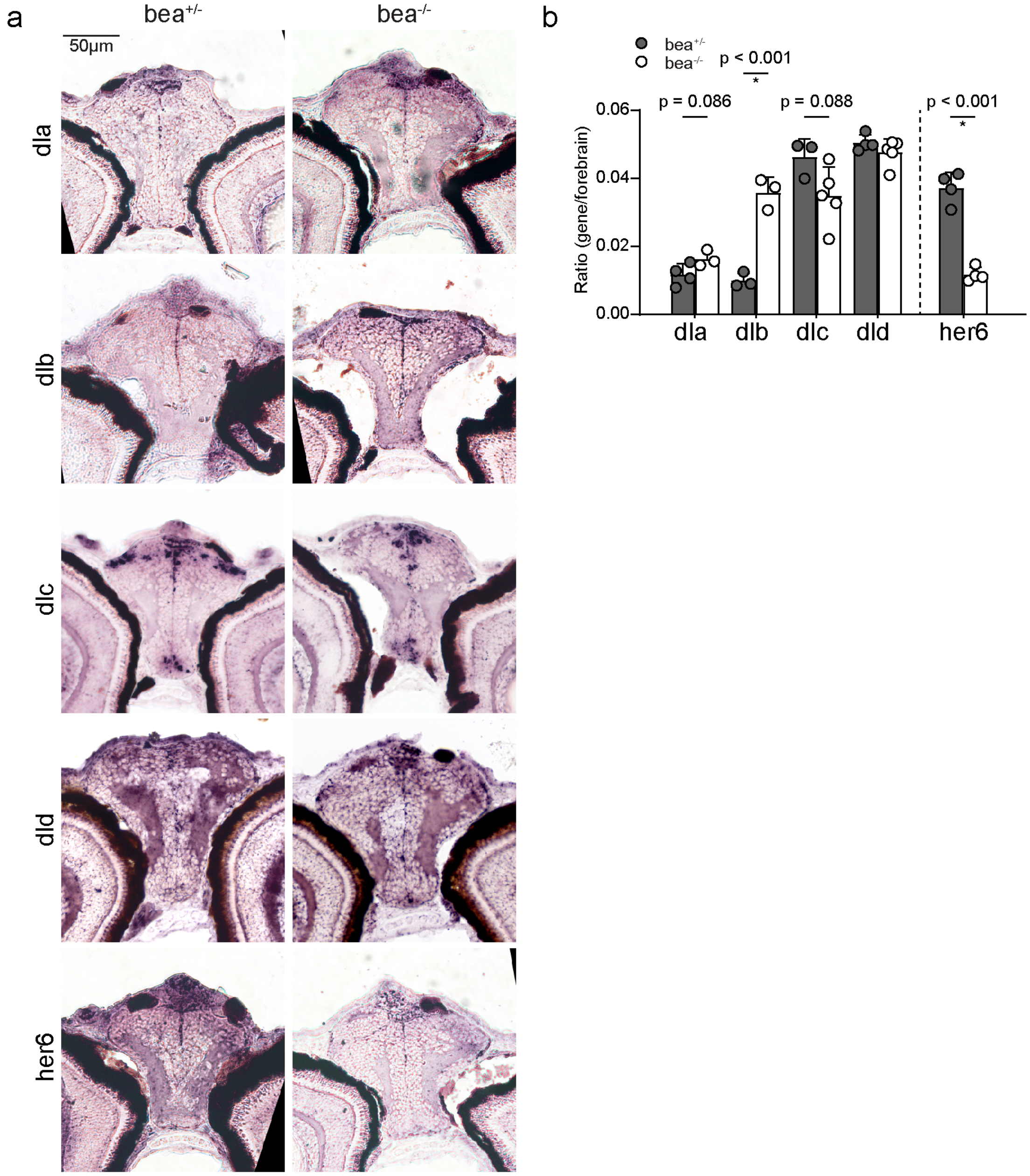

Fig. S4. The expression of *delta* genes and the effector gene of Notch signaling in *dlc* mutant fish

(a-b) In situ hybridization and quantitative analysis of larval zebrafish with *dlc* mutant at 5 dpf using probes targeting delta genes, including *dla-d* and a downstream target of the Notch signaling pathway, *her6*. Scale bars are indicated. Error bars represent standard deviation, and samples are shown in dots. * Significance with P value < 0.001 by student's t-test.

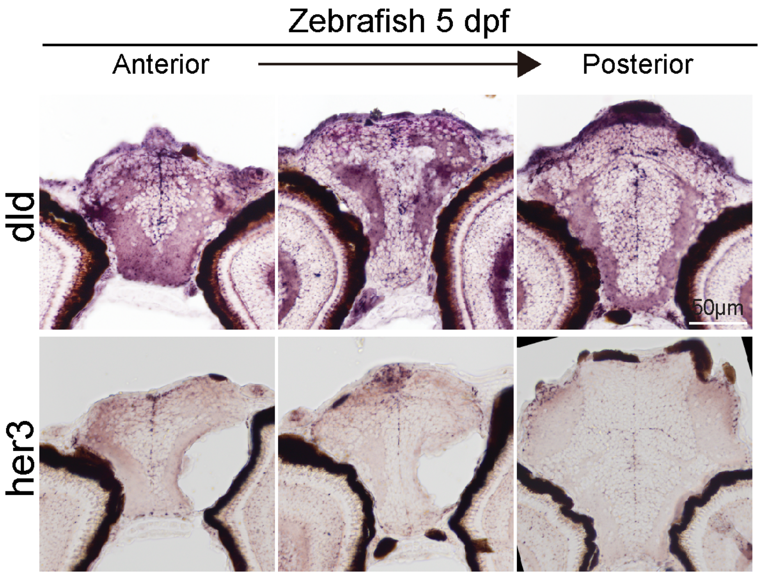

Fig. S5. *dld* and *her3* expression in the forebrain of larval zebrafish at 5 dpf
In situ hybridization of *dld and her3* in the developing forebrain of 5 dpf larval zebrafish using probes specifically targeting *dld* and *her3* mRNA. Scale bars are indicated.

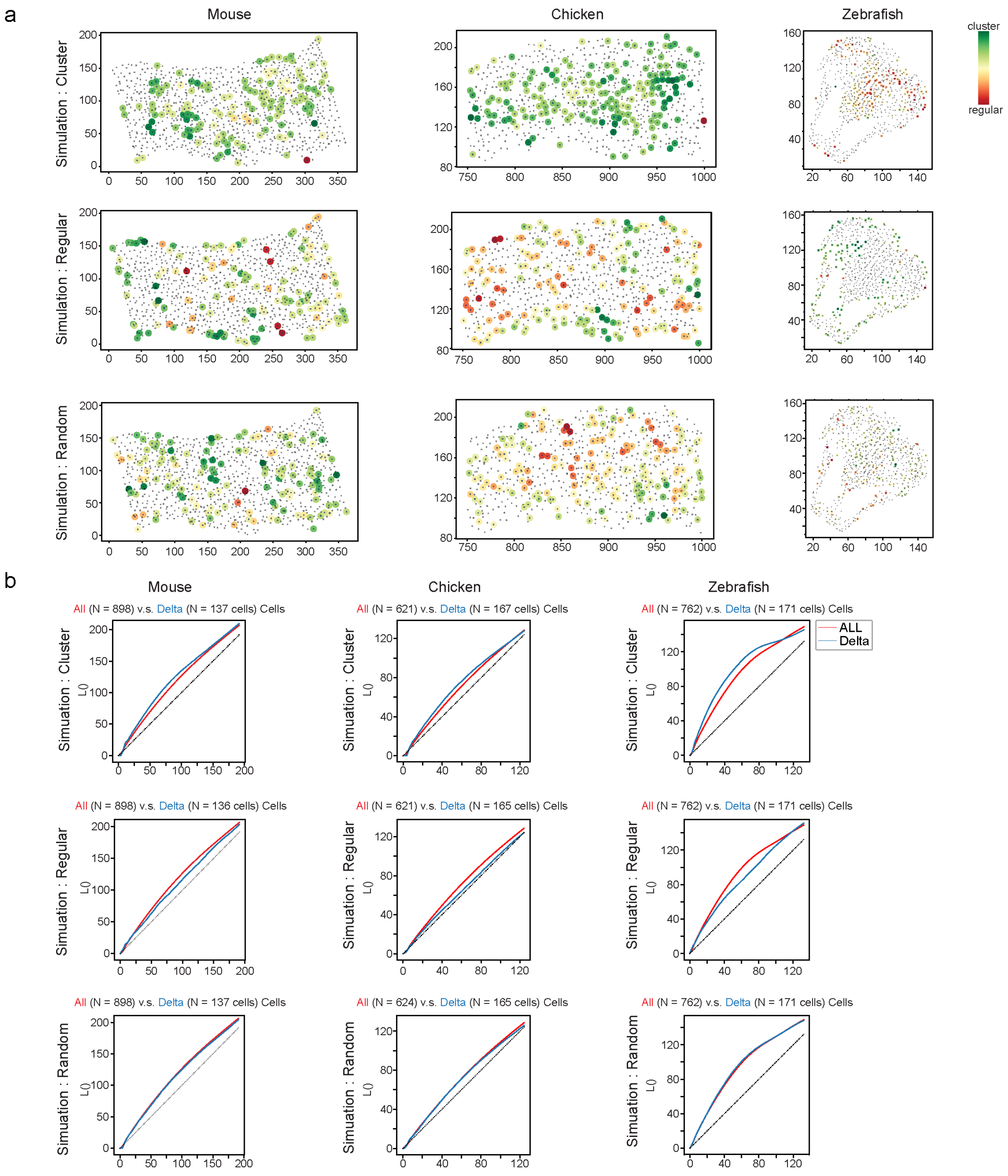

Fig. S6. The simulation of clustering, regularity, and randomness pattern of *Delta/Dll1*-positive cells in mice, chickens, and zebrafish developing pallium

(a) Simulations of Delta spatial expression in clustering, regularity, and randomness manners on the coordinates of all cells.

(b) Simulated L() analysis of the distribution of *Delta/Dll1*-positive cells

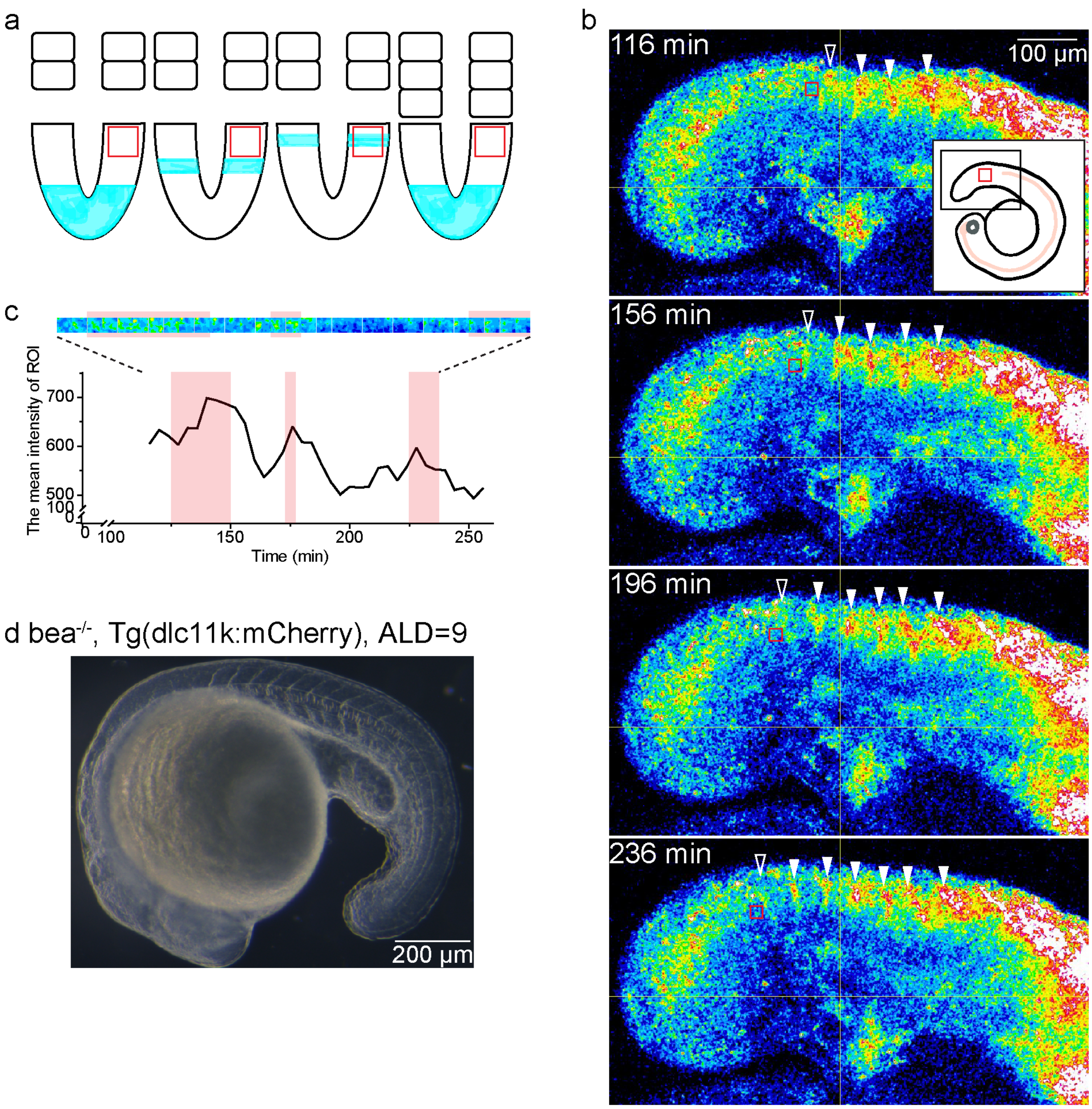

Fig. S7. PSM live imaging of dlc-mCherry transgenic fish showing rescued oscillatory expression during early somitogenesis

(a) The schematic diagram illustrating the oscillatory expression of DeltaC in PSM. The blue color represents the expression of DeltaC protein. Red squares indicate the position of the anterior PSM.

(b) The images showed the signal intensity of dlc-mCherry during the 17 to 20 somite stage. The close arrowheads indicate the boundaries of segmented somites, and the open arrowheads indicate the boundaries of the newly formed somites. Red squares (12.45*12.45 µm) indicate the position of the anterior PSM we quantified.

(c) The montage shows serial images of signal intensity in the red squares as shown in (B) with a 4-minute interval. The line chart shows the periodic expression of the dlc-mCherry signal in the anterior PSM in 140 minutes. The light red boxes represent the peak regions. The corresponding

timeframe of light red boxes from the left to the right are 124-152 min, 172-176 min, and 224-236 min.

(d) The lateral view of *dlc* mutant with dlc-mCherry embryo at the 18-somite stage. ALD, anterior limited defect. Scale bars are indicated.

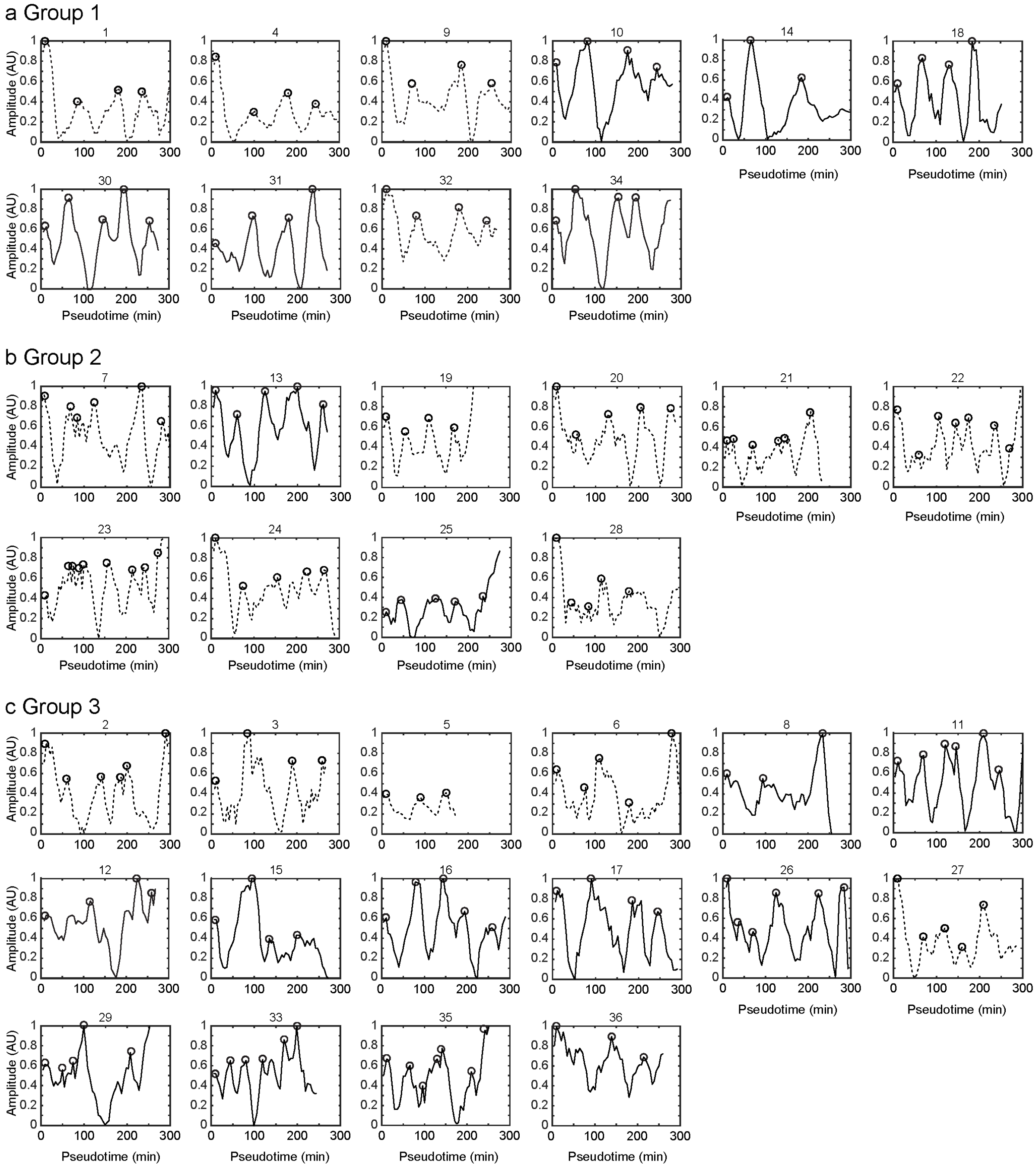

Fig. S8. Dynamic dlc-mCherry signals in the dorsal telencephalon cells over a 300-minute period
(a) Line charts show the amplitude of mCherry signals in the cells in group 1 after the 1st peak alignment.
(b) Line charts show the amplitude of mCherry signals in the cells in group 2 after the 1st peak alignment.
(c) Line charts show the amplitude of mCherry signals in the cells in group 3 after the 1st peak alignment.

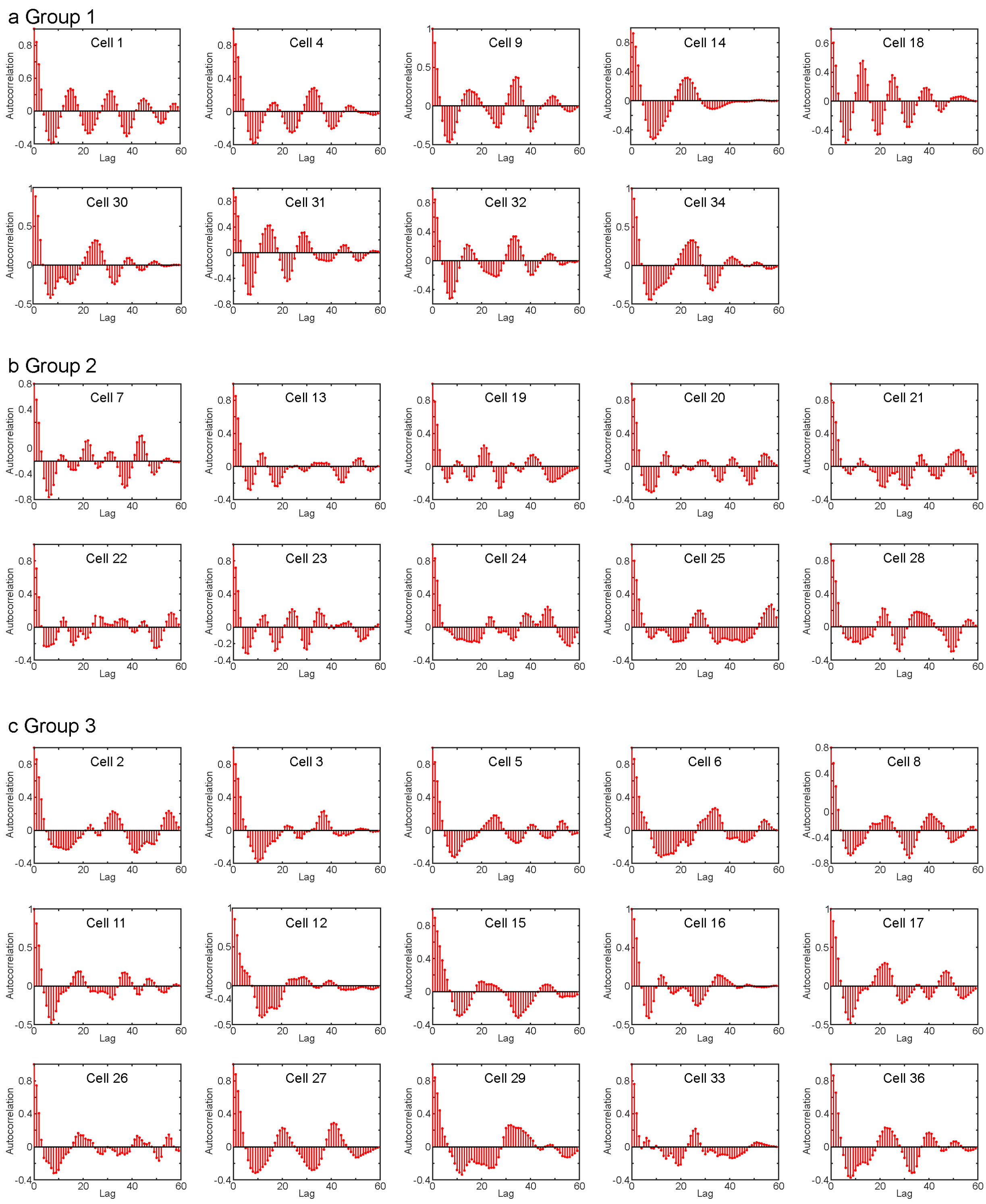

Fig. S9. Autocorrelation results of corrected dlc-mCherry signals in the dorsal telencephalon cells over a 300-minute period

Curve diagrams show the autocorrelation results of mCherry signals in the cells in group 1 (a), group 2 (b) and group 3 (c).

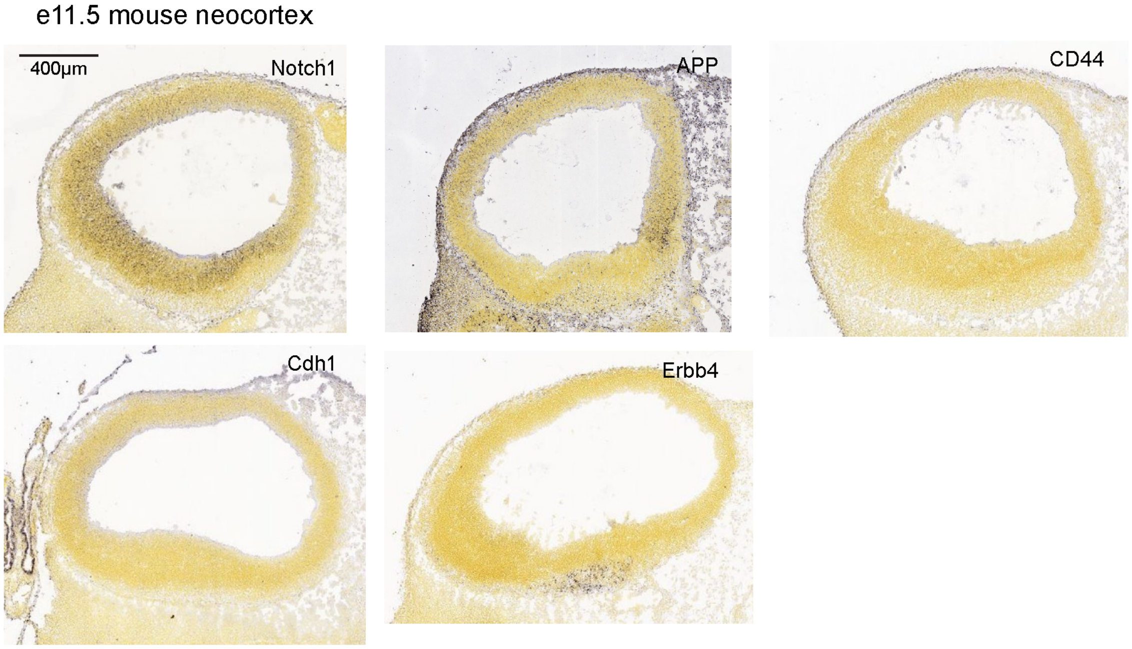

Fig. S10. Expression pattern of the targets of γ-secretase in the developing neocortex at E11.5
The expression patterns of Notch1, APP, CD44, Cdh1, and Erbb4 in the developing neocortex at E11.5 were adapted from the Allen Brain Atlas (https://portal.brain-map.org).

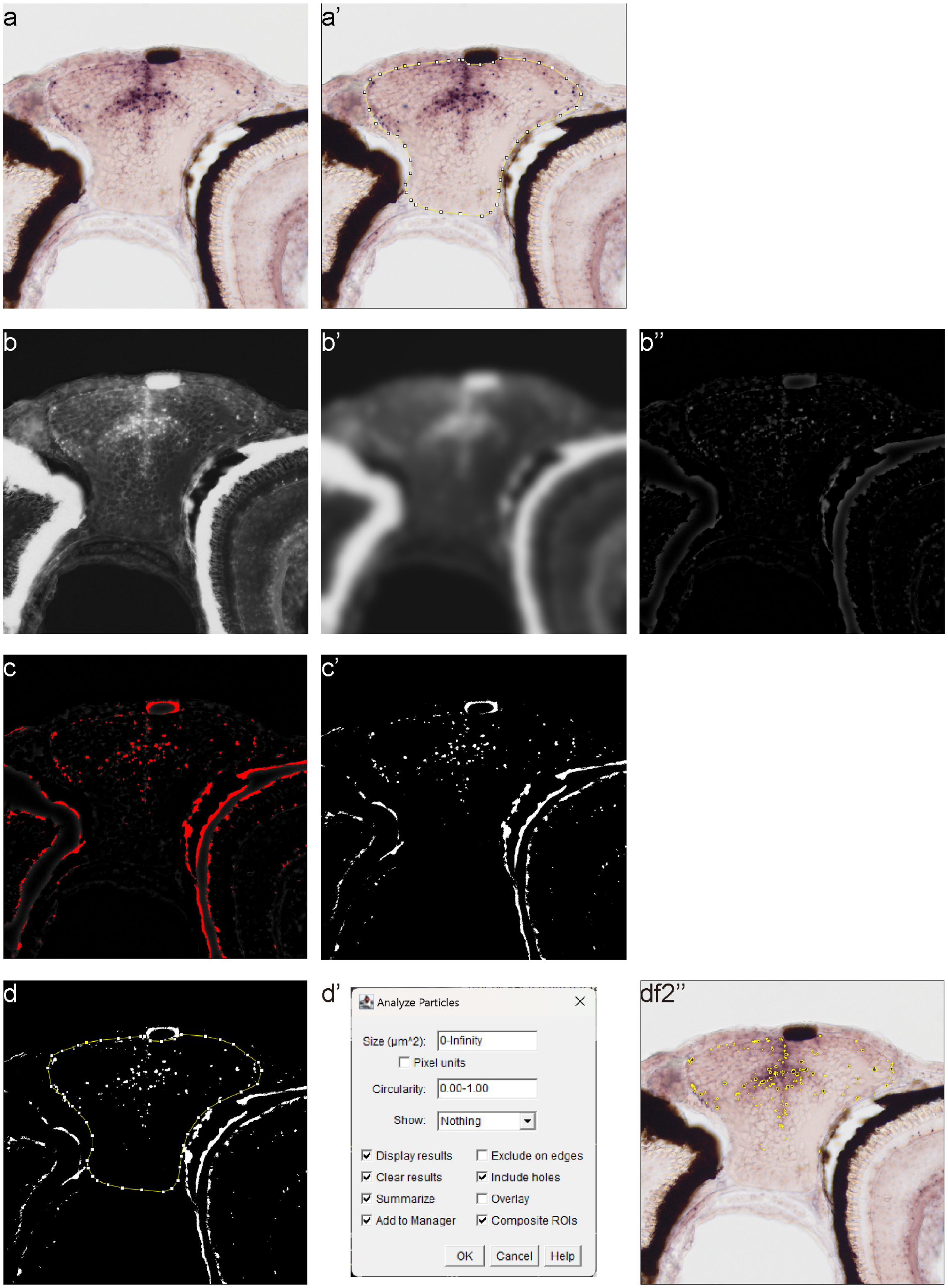

Fig. S11. The procedure of imaging processing for ISH results

(a) The region of the forebrain was identified and selected.

(b) Before quantifying the positive signals, the original image was converted into an 8-bit black-and-white image.
(b') Gaussian background subtraction was applied using a 3-5 μm radius.
(b") The results of subtracting Fig. S10B from Fig. S10B'.
(c-c') The threshold was set to ensure that positive signals were included.
(d) The region of the forebrain from Fig. S10A was applied.
(d') The setting of particle analysis
(d'') Overlapping the results of Particle Analysis to the original ISH image.

Table S1. Primers for ISH probe preparation

Primer	Sequence
Mouse Dll1_forward	GGAGAGCTCATTCCCCTTCGGCTTCAC
Mouse Dll1_reverse	TTCCCGCGGCGAGGTCCACACACTTG
Mouse Hes1_forward	CTCTGGGGACTGAGAAGA
Mouse Hes1_reverse	GGGACTTTACGGGTAGCA
Chicken Dll1_forward	GGCCGCGGTTTGTGTGTGATGAGCAC
Chicken Dll1_reverse	CCCGGGGCTTTTCACAGTTGAACCCAG
Chicken Hes1_forward	GGCCGCGGCCGACATCCTGGAGATGA
Chicken Hes1_reverse	CCCGGGGCTGGTACAAAGGCACAATCC
Turtle Dll1_forward	GGCCGCGGGTGGCAAGGGCGTTATTGTG
Turtle Dll1_reverse	CCCGGGGCGACAACAATAGCAGCGCACC
Turtle Hes1_forward	GGCCGCGGCACCCACTATATTCCCTCTTTC
Turtle Hes1_reverse	CCCGGGGCCTGACGCCACAGATCAATAG
Zebrafish her3_forward	GGCCGCGGCACGCTGGTTACAGAAGTTGTC
Zebrafish her3_reverse	CCCGGGGCTACACAGTGCATGAGAAGCAAAA
Zebrafish her6_forward	GGCCGCGGGCATGACACAGATCAACGCC
Zebrafish her6_reverse	CCCGGGGCTGGCATCACAACGTGGAAAA
Zebrafish sox2_forward	ACTTCTGTCCGAGAGCGAGA
Zebrafish sox2_reverse	TCGTGCCGTTAATCGTCGTA
Zebrafish elavl3_forward	GGCCGCGGGGCTGGTGCATCTTCGTCTAC
Zebrafish elavl3_reverse	CCCGGGGCGCTCCCCCTGCATGTTAAAG
Zebrafish eomesa_forward	GGCCGCGGCGCCCTCTCCTACTGACTCT
Zebrafish eomesa_reverse	CCCGGGGCGGCTGGTGTAGAAGGCGTAA
